# Antioxidant and Anti-inflammatory Activity of Eugenol, Bis-eugenol, and Clove Essential Oil: An In Vitro Study

**DOI:** 10.1021/acsomega.5c04146

**Published:** 2025-07-10

**Authors:** Eduarda Pires Costa, Manoela Maciel dos Santos, Rosinéa Aparecida de Paula, Danilo Aniceto da Silva, Renata Pereira Lopes, Robson Ricardo Teixeira, Reggiani Vilela Gonçalves

**Affiliations:** 1 Department of General Biology, 28120 Federal University of Viçosa, Avenida PH Rolfs, s/n, Campus Universitário, Viçosa, MG 36570-900, Brazil; 2 Department of Animal Biology, 28120 Federal University of Viçosa, Avenida PH Rolfs, s/n, Campus Universitário, Viçosa, MG 36570-900, Brazil; 3 Department of Chemistry, 28120 Federal University of Viçosa, Avenida PH Rolfs, s/n, Campus Universitário, Viçosa, MG 36570-900, Brazil; 4 Department of Animal Science, Plants for Human Health Institute, North Carolina State University, Kannapolis, North Carolina 28081, United States

## Abstract

Different therapeutic approaches, particularly those involving plant-derived compounds, have been explored for treating inflammatory diseases. This study evaluated the antioxidant and anti-inflammatory properties of eugenol (EU) in its pure form, bis-eugenol (BIS), and clove essential oil (OE) at different concentrations (5, 10, and 25 μg/mL). Chemical analysis confirmed that OE contains a high proportion of eugenol (45–90%), with the presence of eugenol acetate differentiating it from pure eugenol, while bis-eugenol was successfully synthesized and characterized with a 97% yield. Antioxidant activity assessed by 2,2-diphenyl-1-picrylhydrazyl (DPPH) and ferric reducing antioxidant power (FRAP) assays showed that 25 μg/mL of each compound effectively neutralized free radicals and reduced ferric ions, with DPPH radical scavenging of about 80% and FRAP values ranging from 200 to 300 μM Fe^2+^ equivalents. In RAW 264.7 macrophages, the extracts were noncytotoxic and maintained above 70% cell viability under H_2_O_2_-induced oxidative stress, with BIS25 showing the highest protective effect. Regarding cellular mechanisms, OE25 preserved superoxide dismutase (SOD) and catalase activity, while BIS10 and BIS25 significantly reduced catalase activity. EU10, EU25, and OE25 led to notable glutathione activity depletion. BIS25 was the only compound to significantly reduce nitric oxide production. All extracts downregulated toll-like receptor 4 (TLR-4) expression. BIS10 induced NRF2, and IL-10 increased with BIS10 and OE10. Tumor necrosis factor alpha (TNF-α) levels decreased across all BIS and OE concentrations. Our findings indicate that bis-eugenol exhibited the most pronounced antioxidant and anti-inflammatory effects among the compounds tested. Its chemical structure appears to confer greater stability and reduce the generation of phenoxy radicals, which may account for its superior efficacy in cellular protection and inflammatory modulation, especially through activation of the NRF2 pathway. Notably, bis-eugenol was the only compound that simultaneously suppressed TLR4/nuclear factor kappa B (NF-κB) pathways while upregulating NRF2 and IL-10, suggesting that its mechanism of action involves both direct inflammatory inhibition and the activation of endogenous protective pathways. However, further studies are needed to elucidate the influence of its molecular structure on these mechanisms and to confirm its primary role in mediating these effects.

## Graphical abstract

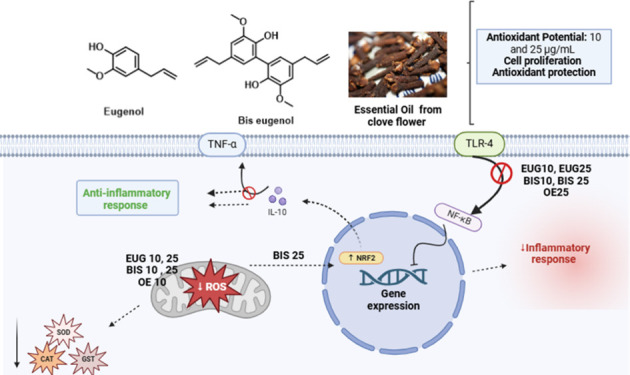

## 1 Introduction

Inflammation is the body’s biological response to tissue damage or infection, characterized by phenomena such as increased blood flow, leukocyte infiltration, and the release of biochemical mediators that help repair tissues and eliminate pathogens.^1^ In this context, immune cells, such as macrophages, play a crucial role by releasing cytokines, chemokines, and reactive oxygen species (ROS), which act as mediators of the inflammatory response.^2^ While the controlled generation of ROS is physiologically important for signaling pathways related to cell proliferation and immune responses,^3^ excessive ROS production can overwhelm the body’s antioxidant defenses, leading to oxidative stress.^4^ This imbalance promotes the oxidation of biomolecules such as lipids, proteins, and DNA, compromising cell function and contributing to tissue damage.^5^ Moreover, ROS can act as secondary messengers that activate pro-inflammatory transcription factors like NF-κB, triggering the release of further inflammatory mediators and amplifying the inflammatory response in a positive feedback loop.^6^

Currently, different therapeutic interventions and their mechanisms of action, both cellular and extracellular, have been investigated for a better understanding of this process, and different compounds have been used to treat inflammatory diseases, especially plant extracts, exhibiting antioxidant and anti-inflammatory actions. These effects are often attributed to the modulation of redox metabolism and the suppression of oxidative stress, highlighting their potential in the development of novel approaches for inflammation control and tissue repair.^7^ Studies show that compounds from some natural extracts not only have antioxidant capacity but are also neuroprotective, immunomodulatory, anti-inflammatory, regenerative, and healing, showing high therapeutic potential.^8,9^

Eugenol (4-allyl-2-methoxyphenol) is an aromatic phenolic compound widely found in various plants from the Lamiaceae, Lauraceae, Myrtaceae, and Myristicaceae families. It is one of the major constituents of clove (*Syzygium aromaticum* (L.) Myrtaceae) essential oil and is commonly used as a flavoring agent in cosmetics and food products.^10^ This compound has attracted considerable interest in the scientific community due to its broad spectrum of biological activities, making it a promising candidate for the development of therapeutic agents targeting inflammatory and oxidative stress-related diseases.^11^ However, it is important to note that eugenol may exhibit toxicity at high concentrations. In addition, some compounds structurally related to eugenol also demonstrate antioxidant, anti-inflammatory, antiviral, antifungal, antibacterial, anticancer, antidiabetic, and neuroprotective activities.^12^ Bis-eugenol, an ortho-dimer of eugenol found in low concentrations in *Syzygium aromaticum* and certain natural products, has exhibited higher anti-inflammatory and antioxidant activities than eugenol.^13^ However, there are no studies directly comparing eugenol, bis-eugenol, and clove essential oil-related compounds in RAW 264.7 macrophages, hindering a comprehensive understanding of their differences in terms of efficacy and mechanisms of action. Macrophages are key cells in acute inflammation, responsible for producing ROS and pro-inflammatory cytokines. We used the RAW 264.7 cell line due to its high plasticity, which allows it to adapt to environmental stimuli such as bioactive compounds. This model is well-suited for examining how such compounds influence oxidative stress and the inflammatory process, especially during the respiratory burst.^14^

Considering the promising antioxidant and anti-inflammatory properties of eugenol and its derivatives, the aim of this study is to compare the antioxidant and anti-inflammatory effects of eugenol, bis-eugenol, and clove essential oil at varying concentrations in RAW 264.7 macrophages, with a focus on elucidating potential mechanistic differences and therapeutic relevance. Based on the hypothesis that bis-eugenol, due to its dimeric structure, may exhibit enhanced bioactivity compared to eugenol and the crude essential oil, the study seeks to evaluate the relative efficacy of these compounds in modulating oxidative stress and inflammatory markers. This comparative analysis is intended to provide insights into the structure–activity relationships of these phenolic agents and support the development of more effective natural anti-inflammatory and antioxidant formulations.

## 2 Materials and Methods

### 2.1 Generalities of Eugenol and Clove Essential Oil

Infrared (IR) spectra were acquired using an ALPHAA II spectrophotometer (Bruker, Billerica, Massachusetts, USA) equipped with an attenuated reflectance accessory (ATR) over the region of 400–4000 cm^–1^ with 64 scans and 4 cm^–1^ of spectral resolution. Nuclear magnetic resonance spectra were recorded on Bruker 400 MHz AvanceCore spectrometer (Bruker, Billerica, MA, USA) or Varin Mercury 300 (Varian, Palo Alto, CA, USA) using deuterated chloroform (CDCl_3_) as solvent. Coupling constants (*J*) were expressed in Hertz (Hz) and chemical shift (δ) in ppm. Signal multiplicities were denoted as multiplet (m), singlet (s), doublet (d), apparent of doublet of doublets of triplets (ddt_apt_).

#### 2.1.1 Chemicals

Dichloromethane was purchased from Êxodo Científica (Sumaré, São Paulo City, São Paulo State, Brazil). Eugenol and octamethylcyclotetrasiloxane were purchased from Sigma-Aldrich (St. Louis, MO, USA) and used as received. Amonium hydroxide, HCl (37% w/v), potassium ferricyanide, and acetone were purchased from F. Maia (Belo Horizonte, Minas Gerais State, Brazil).

#### 2.1.2 Extraction of Clove Essential Oil

The clove essential oil (EO) was extracted according to an adapted procedure based on previous studies.^15^ Clove samples were purchased from a local market in Viçosa, Minas Gerais, Brazil, and used as received, without any prior treatment. For extraction, 40.0 g of cloves were weighed into a round-bottom flask, and 500 mL of type 1 water was added. The mixture was heated at 100 °C for 3 h without agitation, allowing the steam to form and condense. The distillate (hydrolate) was collected and then subjected to three sequential liquid–liquid extractions with dichloromethane (100 mL for each step). The combined organic layers were concentrated by evaporating the solvent under reduced pressure at 22 °C for 40 min using a rotary evaporator. The obtained EO was stored in an amber glass vial protected from light and kept refrigerated at 4 °C. The oil yield was calculated according to eq 1:

$$\mathrm{yield}\;(\%)=(m_{0}/m_{1})\times 100$$ 1

where *m*_0_ represents the mass of extracted EO and *m*_1_ is the mass of clove used.

#### 2.1.3 Quantifying Eugenol Content in Clove Essential Oil by Nuclear Magnetic Resonance (NMR)

The hydrogen nuclear magnetic resonance (^1^H NMR) spectra were recorded using a Bruker 400 MHz AvanceCore spectrometer equipped with a 5 mm broadband inverse detection (BBI) probe, a field gradient generator unit, and a temperature control unit. Deuterated chloroform without tetramethyl silane (TMS) as the solvent and 98% octamethylcyclotetrasiloxane as the internal standard were utilized. The 45° pulse (pw45) was determined for all samples using the calibration sequence implemented by Bruker TopSpin 4.3.0 software. The experiment was conducted at a temperature of 25 °C. The longitudinal relaxation time (T1) was experimentally determined using the inversion recovery sequence for all hydrogen nuclei in the sample. The highest *T*1 value obtained for the quantification of eugenol in the essential oil samples, including the internal standard, was 7 s. The number of scans (ns) was 16, the acquisition time (aq) corresponded to 3.9976959 s, and the delay time (d1) was 35 s (5 × *T*1). The analysis was performed in triplicate (n = 3). For the quantification of eugenol, eq 2 was used:

$$P_{\mathrm{Eug}}=\frac{I_{\mathrm{Eug}}}{I_{\mathrm{OMCTS}}}\times \frac{N_{\mathrm{OMCTS}}}{N_{\mathrm{Eug}}}\times \frac{M_{\mathrm{Eug}}}{M_{\mathrm{OMCTS}}}\times \frac{m_{\mathrm{OMCTS}}}{m}\times P_{\mathrm{OMCTS}}$$ 2

Where: *P*_Eug_ = percentage of eugenol in the essential oil; *I*_Eug_ = area measured by integration of the desired signal in the ^1^H NMR of eugenol (3.8 ppm); *I*_OMCTS_ = area measured by the integration of octamethylcyclotetrasiloxane (1000); *N*_OMCTS_ = number of hydrogens of octamethylcyclotetrasiloxane (24); *N*_Eug_ = number of hydrogens of the desired signal of the eugenol (Methoxy, 3H); *M*_Eug_ = molar mass of eugenol (164.20 g·mol^–1^); *M*_OMCTS_ = molar mass of the internal standard (296.62 g·mol^–1^); *m*_OMCTS_ = mass of octamethylcyclotetrasiloxane (0.00658 g); *m* = mass of the essential oil sample (0.03682 g); *P*_OMCTS_ = purity of octamethylcyclotetrasiloxane (98%) informed by the supplier (Sigma-Aldrich, St. Louis, MO, USA).

In the Supporting Information, the ^1^H NMR spectra of clove essential oil, recorded for the quantification of eugenol, are presented in Figure S3A–C. For comparison, the ^1^H NMR spectrum of pure eugenol is shown in Figure S2. Finally, Figure S4 presents the COSY analysis of clove essential oil, confirming that the methoxy group’s signal remains distinct from other signals. NMR analysis integrated the methoxy group’s hydrogen signals for the quantification process.

#### 2.1.4 Gas Chromatography/Mass Spectrometry (GC-MS) Analysis of the Clove Essential Oil

The clove essential oil was dissolved in dichloromethane for characterization by gas chromatography coupled with mass spectrometry (GC-MS) employing the GC-MSQP2010 Ultra spectrometer equipment, Shimadzu, Kyoto, Japan. The conditions used were: He as the carrier gas, with a flow of 1.47 mL min^–1^; injector temperature of 300 °C at a split ratio of 1:50; fused silica capillary column (30 m × 0.25 mm) containing RX1-1MS stationary phase (0.25 μm film thickness). The oven temperature was programmed as follows: initial temperature of 40 °C for 1 min. Then, the temperature was increased with a heating rate of 5 °C min^–1^ until reaching 240 °C and remained for 2 min; Thereafter, the temperature was increased to 290 °C with a heating rate of 5 °C min^–1^. The total time of the analysis was 54 min. The compounds were identified according to the similarity of the library database (Wiley 7, NIST 05, 1168).

### 2.2 Generalities of Bis-Eugenol

The melting point was determined using an MQAPF-302 apparatus (Micro Química, Cotia, São Paulo, Brazil) and were not corrected. Infrared (IR) spectra were acquired using the attenuated total reflectance (ATR) technique on a Varian 660-IR instrument equipped with a GladiATr accessory (Varian, Palo Alto, CA, USA) in the region of 4000–500 cm^–1^. Nuclear magnetic resonance spectra were recorded on Varian Mercury 300 MHz (Varian, Palo Alto, CA, USA). Chloroform (CDCl_3_) served as deuterated solvent. Coupling constants (*J*) were expressed in Hertz (Hz) and chemical shift (δ) in ppm. Signal multiplicities were denoted as singlet (s), doublet (d), doublet of doublets of triplets (ddt).

#### 2.2.1 Experimental Procedure for the Preparation of Bis-Eugenol

In a 100 mL round-bottom flask containing a solution of eugenol (1.00 g, 6.10 mmol, 1.00 equiv) in an acetone-distilled water mixture (2:1 v/v, 30 mL), 18 mL of aqueous NH_4_OH was added, and the mixture was stirred for 10 min. Subsequently, a saturated aqueous solution of K_3_Fe­(CN)_6_ (2.00 g, 6.10 mmol, 1.00 equiv) was added dropwise over approximately 5 h. Afterward, 18 mL of aqueous NH_4_OH was added, and the reaction mixture was stirred for 16 h at room temperature and then neutralized with HCl (37% w/v aqueous solution). A solid precipitate was formed, which was filtered, washed with distilled water, and dried under reduced pressure. The crude product was crystallized from dichloromethane and obtained as a pale yellow solid with a 97% yield (0.969 g, 3.05 mmol). The following data confirmed the structure of bis eugenol.

TLC: R_f_ = 0.38 (hexane-ethyl acetate 2:1 v/v).

Melting point: 104–105 °C [Literature: 106–107 °C].^1^

IR (ATR) ${\overset{-}{v}}_{\max}$ : 3275, 2999, 2948, 2908, 2881, 2830, 1699, 1636, 1508, 1461, 1420, 1377, 1326, 1248, 1183, 1140, 1052, 998, 947, 899, 841, 801, 732, 668, 612, 557, 501, 436.

^1^H RMN (300 MHz, CDCl_3_) δ: 3.36 (d, 4H, *J* = 6.7 Hz), 3.87 (s, 6H), 5.16–4.99 (m, 4H), 5.45 (s, 2H, OH), 5.98 (ddt_ap_, 2H, *J*_*trans*_ = 16.8 Hz, *J*_*cis*_ = 10.2 Hz, *J* = 6.6 Hz), 6.69 (d, 2H, *J* = 2.0 Hz), 6.78 (d, 2H, *J* = 2.0 Hz).

RMN de ^13^C (75 MHz, CDCl_3_) δ: 40.2, 55.9, 110.7, 115.6, 123.2, 126.2, 131.0; 138.0; 142.8, 148.4.

The infrared (IR) and NMR (^1^H and ^13^C) used in the characterization of bis eugenol are found in the Supporting Information (Figures S8–S10).

### 2.3 Antioxidant Analysis

#### 2.3.1 DPPH Radical Scavenging Assay

To evaluate the antioxidant capacity of the extracts, the DPPH radical scavenging assay was performed according to the method described by Herald et al., 2022.^16^ The antioxidant activity is evaluated based on the reduction of the DPPH radical, leading to the formation of diphenyl-picryl-hydrazine, a yellow-colored compound, in a reaction that stabilizes after 30 min. Eugenol, bis eugenol, and clove essential oil were evaluated at concentrations of 5 μg/mL, 10 μg/mL, and 25 μg/mL, prepared in methanol.Ascorbic acid (50 μg/mL, diluted in methanol) was employed as the positive control for evaluating antioxidant capacity. For the assay, 50 μL of each extract or ascorbic acid solution was mixed with 250 μL of a freshly prepared 0.01 mM DPPH solution. The reaction mixtures were incubated in the dark for 30 min to allow the scavenging reaction to occur. Subsequently, the absorbance was measured at 517 nm using a microplate spectrophotometer. The percentage of DPPH radical scavenging activity was determined according to eq 3.

$$\mathrm{DPPH}\;\mathrm{inhibition}\;(\%)=\frac{\mathrm{Abs}\;\mathrm{DPPH}/\mathrm{methanol}-\mathrm{Abs}\;\mathrm{extracts}}{\mathrm{Abs}\;\mathrm{DPPH}/\mathrm{methanol}}\times 100$$ 3

#### 2.3.2 Ferric Reducing Antioxidant Power (FRAP) Assay

The total antioxidant capacity of the samples was assessed by the Ferric Reducing Antioxidant Power (FRAP) assay, following the procedure described by Benzie and Strain,^17^ using 2,4,6-Tris­(2-pyridyl)-s-triazine (TPTZ) as the complexing agent. This method relies on the reduction of the ferric–TPTZ complex (Fe^3+^–TPTZ) to its ferrous form (Fe^2+^–TPTZ) under acidic conditions. For the reaction, 10 μL of each sample (eugenol, bis-eugenol, or clove essential oil) at concentrations of 5, 10, and 25 μg/mL was added to 190 μL of freshly prepared FRAP reagent. The working FRAP solution was composed of 25 mL of acetate buffer (300 mM, pH 3.6), 2.5 mL of 10 mM TPTZ solution, and 2.5 mL of 20 mM FeCl_3_·6H_2_O solution.The increase in absorbance at 593 nm was measured to determine the Fe^3+^ + −TPTZ complex reduction by antioxidants in the samples. The reducing capacity was quantified using a standard curve prepared from serial dilutions of FeSO_4_·7H_2_O starting at one μmol/L. The results were expressed as FRAP values (μmol). A 50 μg/mL concentration of Ascorbic acid was used as the reference standard for comparison. The experiments were performed at least in triplicate.

### 2.4 In Vitro Analysis

#### 2.4.1 Cell Viability Analysis

Cell proliferation assays performed on RAW 264.7 macrophages cells were evaluated using the 3-(4,5-dimethylthiazol-2-yl)-2,5-diphenyltetrazolium bromide (MTT) reduction method.^18^ Macrophage cells were seeded at a density of 2 × 10^4^ cells in 200 μL per well in 96-well plates, using DMEM as the culture medium supplemented with 10% fetal bovine serum (FBS), and incubated in a humidified incubator at 37 °C with 5% CO_2_ for 24 h. Afterward, the supernatant was removed, and the extracts (eugenol, bis-eugenol, and clove essential oil) were added at 10 μg/mL and 25 μg/mL concentrations. A negative control containing only culture medium was included. The cells were incubated (37 °C, 5% CO_2_) for 22 h. Subsequently, 50 μL of supernatant from each well was removed, and 50 μL of MTT solution (0.5 mg/mL) was added. The cells were further incubated for 1 h. The formation of formazan crystals was observed. Then, the supernatant from each well was removed, and 100 μL of DMSO was added. Absorbance was measured using a Multiskan FC Microplate Reader (Thermo Labsystems, Franklin, MA, USA) set to 570 nm. All treatments were conducted in triplicate wells and the experiments were repeated at least three independent times.

#### 2.4.2 Cell Viability Analysis after H_2_O_2_-Induced Oxidative Stress

Macrophage cells were seeded at a density of 2 × 10^4^ cells per well in a 96-well plate containing a DMEM culture medium supplemented with 10% FBS. The cells were incubated at 37 °C with 5% CO_2_ for 24 h and subsequently exposed to the Eugenol and Bis eugenol extracts and clove essential oil at concentrations of 10 μg/mL and 25 μg/mL. After 24 h, the cells were exposed to 1.5 mM H_2_O_2_ for 2 h. Cell viability assessment was performed using the MTT assay described in the previous section. All treatments were conducted in triplicate wells and the experiments were repeated at least three independent times.

#### 2.4.3 Evaluation of Antioxidant Enzyme Activity and Oxidative Stress Products

RAW 264.7 macrophages were seeded at a density of 1 × 10^6^ cells per well in 96-well plates containing DMEM supplemented with 10% FBS. Cells were incubated for 24 h at 37 °C in a humidified atmosphere with 5% CO_2_. After incubation, the medium was removed, and the cells were treated with eugenol, bis-eugenol, or clove essential oil at final concentrations of 10 μg/mL or 25 μg/mL for 24 h. Following treatment, cells were challenged with 1.25 mM hydrogen peroxide (H_2_O_2_) for 3 h. Afterward, the culture medium was discarded, and the cells were lysed in PBS containing 1% Triton X-100 for subsequent analysis of catalase (CAT), superoxide dismutase (SOD), and glutathione S-transferase (GST) activities. CAT activity was determined based on the degradation of H_2_O_2_, according to Aebi (1984), with modifications.^19^ Briefly, 5 μL of cell lysate was added to wells containing 100 μL of 20 mM H_2_O_2_ solution. After 3 min, the reaction was stopped with 150 μL of ammonium molybdate, and residual H_2_O_2_ was measured spectrophotometrically at 374 nm using a standard calibration curve (0.078–20 mM). SOD activity was quantified by mixing 30 μL of sample with 99 μL of phosphate buffer (0.2 M, pH 8.0), 6 μL of MTT (1.25 mM), and 15 μL of pyrogallol (100 μM). The reaction was incubated at 40 °C for 15 min and absorbance was recorded at 540 nm. Controls and blanks were prepared similarly, omitting the sample or pyrogallol as appropriate. GST activity was assessed using the method described by Habig et al. (1974)^20^ with modifications. The reaction mixture contained 970 μL of potassium phosphate buffer (pH 7.0), 10 μL of CDNB, 10 μL of reduced glutathione (GSH), and 10 μL of the cell lysate. Enzyme activity was monitored by measuring absorbance at 340 nm at intervals of 0, 30, 60, and 90 s. Nitric oxide (NO) production was indirectly quantified by measuring nitrite levels via the Griess reaction. A standard curve was generated with sodium nitrite (0–0.25 mM). For each sample, 50 μL of culture supernatant or standard solution was mixed with equal volumes of sulfanilamide and *N*-(1-naphthyl)­ethylenediamine solutions (1:1) and incubated in the dark for 10 min to allow formation of the azo dye. Absorbance was measured at 570 nm.

#### 2.4.4 mRNA Extraction and Quantitative Real-Time qPCR

RAW 264.7 macrophages were seeded in 6-well culture plates at a density of 2.5 × 10^5^ cells per well in DMEM supplemented with 10% fetal bovine serum (FBS) and incubated for 24 h at 37 °C in a humidified 5% CO_2_ atmosphere. After this initial incubation, cells were treated with eugenol, bis-eugenol, or clove essential oil at final concentrations of 10 μg/mL and 25 μg/mL for 24 h. Following treatment, cells were stimulated with lipopolysaccharide (LPS, 10 μg/mL) for 4 h to induce an inflammatory response. Total RNA was then isolated using TRI Reagent (Sigma-Aldrich), according to the manufacturer’s instructions. The extracted RNA was quantified and assessed for purity using a μDrop Duo Plate in a Multiskan SkyHigh spectrophotometer (Thermo Fisher Scientific). For cDNA synthesis, 1000 ng of total RNA was reverse transcribed using the High-Capacity cDNA Reverse Transcription Kit (Thermo Fisher Scientific). Quantitative real-time PCR (RT-qPCR) was carried out with PowerTrack SYBR Green Master Mix (Thermo Fisher Scientific) on a QuantStudio 3 Real-Time PCR System (Thermo Fisher Scientific).^21^ A total of 1000 ng of extracted RNA was reverse transcribed into cDNA using the High-Capacity cDNA Reverse Transcription Kit (ThermoFisher Scientific). RT-qPCR was performed using PowerTrack SYBR Green Master Mix (ThermoFisher Scientific) in a QuantStudio 3 Real-Time PCR System (ThermoFisher Scientific). Sample normalization was performed based on the ratio between the relative quantity of the target gene and the relative quantity of the reference gene β-actin, whose expression levels were determined using standard curves generated from serial dilutions of cDNA. Primer sequences used for real-time qPCR are listed in Table 1.

**Table 1:** Primer Sequences Used in the Real-Time qPCR Assays

Gene		Sequence (5′-3′)	References
NFK-β	NFKβ-F	GCT GCC AAA GAA GGA CAC GAC A	^22^
	NFKβ-R	GGC AGG CTA TTG CTC ATC ACA G	
TLR-4	TLR4-F	TCT GGG GAG GCA CAT CTT	^23^
	TLR4-R	CTG CTG TTT GCT CAG GAT TC	
NRF-2	NRF2	TAC AGT CCC AGC AGG ACA TGG ATT TG	^24^
	NRF2	GTT TTC GGT ATT AAG ACA CTG TAA TTC GGG AAT GG	
IL-10	IL10-F	TTA ATA AGC TCC AAG ACC AAG G	^25^
	IL10-R	CAT CAT GTA TGC TTC TAT GCA G	
β-actin (ACTB)	β-Actin_F	GTT TTG TTT TGG CGC TTT TG	^26^
	β-Actin_R	AAC TTT GGG GGA TGT TTG CT	

#### 2.4.5 TNF-α Quantification

RAW 264.7 macrophages (2.5 × 10^5^ cells/well) were seeded in 6-well plates with DMEM medium supplemented with 10% FBS and incubated for 24 h. Subsequently, the macrophages were treated with 10 μg/mL and 25 μg/mL of the extracts (eugenol, bis-eugenol, and clove essential oil) and incubated at 37 °C with 5% CO_2_ for 24 h. Afterward, the cells were stimulated with LPS (10 μg/mL) and incubated for 4 h. The supernatant was collected for analysis. According to the manufacturer’s instructions, TNF-α expression was determined using the Mouse TNF-α ELISA kit (Invitrogen, Cat. No. 88-73224). Initially, the microtiter plate was coated with 100 μL of capture antibody diluted in PBS (2 μg/mL) and incubated overnight at 4 °C. Following incubation, the plate was washed three times with PBS-T (PBS + 0.05% Tween-20) and blocked with 200 μL of diluent containing 1% BSA for 1 h at room temperature. Next, recombinant TNF-α standard dilutions were prepared in PBS + 1% BSA, and 100 μL of standards and cell culture samples (5 × 10^5^ cells) were added to each well and incubated overnight at 4 °C. The samples were removed, and the plate was washed three times with PBS-T. Subsequently, 100 μL of detection antibody was added to each well and incubated for 1 h at room temperature. After removing the antibody detection and washing the plate three times with PBS-T, the streptavidin-HRP conjugate was diluted according to the kit instructions, and 100 μL of this reagent was added to each well, followed by a 30 min incubation at room temperature. A 100 μL aliquot of TMB (3,3′,5,5′-tetramethylbenzidine) substrate was added to each well, and the plate was incubated until color development. The reaction was stopped with 100 μL of stop solution, and the absorbance was measured at 450 nm using a plate spectrophotometer.

#### 2.4.6 Statistical Analysis

Statistical analysis was performed using GraphPad Prism, version 8.0. Data normality was assessed using the Kolmogorov–Smirnov test. If the data followed a normal distribution, one-way analysis of variance (One-Way ANOVA) was used for comparisons between experimental groups, followed by Tukey’s multiple comparison test at a 5% significance level.

## 3 Results

### 3.1 Characterization of Clove Essential Oil: GC-MS, ^1^H NMR, and Infrared Spectroscopy Analyses

The essential oil (EO) was extracted from clove flower buds purchased from a local market by hydrodistillation, resulting in 9% w/w. The yellow oil obtained was analyzed by gas chromatography coupled with mass spectrometry (GC-MS), revealing eugenol (82.2%), eugenol acetate (15.8%), and β-caryophyllene (1.0%) as the main constituents. The results of the GC-MS analysis of clove EO are summarized in Table S1 of the Supporting Information. Typically, clove-derived EOs contain 45% to 90% eugenol. In this study, GC-MS analysis determined an eugenol content of 82.2%, consistent with the values reported in the literature.

^1^H NMR characterized the EO obtained. The ^1^H NMR spectrum of pure eugenol (Figure S2, SupportingInformation) shows seven distinct signals corresponding to different hydrogen environments within the eugenol structure. As shown in Figure S3A–C (Supporting Information), these signals are also present in the ^1^H NMR spectra of EO. The eugenol content was quantified as described in the Materials and Methods section, resulting in a value of 86.6 ± 0.27%, consistent with the GC-MS result (82.2%). Figure S4 (Supporting Information) shows the COSY analysis of clove EO, confirming that the signal from the methoxy group remains distinct from the other signals. The hydrogens of the methoxy group were integrated for quantification. The hydrogen assignments for eugenol, the main component of EO, are provided in the Supporting Information (Figure S2). For completeness, the ^13^C NMR spectrum of pure eugenol is also included (Figure S5).

The infrared spectrum of OE (Figure S6, Supporting Information) shows a band at 3514 cm^‑1^, corresponding to the stretching vibration of the OH group present in eugenol. The band at 2946 cm^‑1^ is attributed to the Csp^3^-H stretching, while the band at 1514 cm^‑1^ corresponds to the C=C stretching of the aromatic ring. The bands at 1384 and 1247 cm^‑1^ are associated with CH_2_ deformation vibrations attributed to eugenol and eugenol acetate in the EO. The presence of eugenol acetate is confirmed by the band at 1768 cm^‑1^, which is absent in the IR spectrum of pure eugenol (Figure S7, Supporting Information).

### 3.2 Preparation of Bis-Eugenol from Eugenol

The oxidative coupling reaction of eugenol was performed using potassium ferricyanide as the oxidizing agent, as illustrated in Figure 1. Bis-eugenol was obtained as a yellowish solid with a 97% yield. The product was characterized by NMR and infrared spectroscopy, and the obtained ata were consistent with those reported in the literature (Figure 1). Detailed spectral data, including ^1^H NMR, ^13^C NMR, and IR spectra, are provided in the Supporting Information (Figures S8–S10).

**Figure 1:**
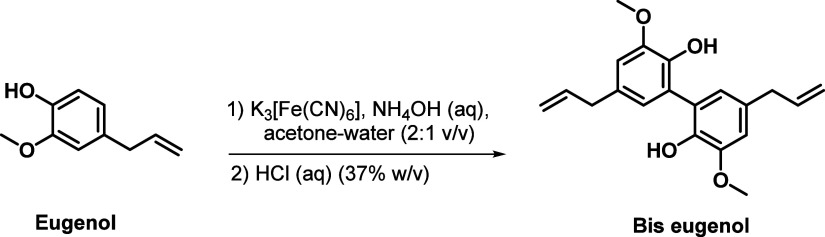
Preparation of bis-eugenol from eugenol via oxidative coupling.

### 3.3 DPPH Radical Scavenging Assay

At the highest tested concentration (25 μg/mL), eugenol (EU25), bis-eugenol (BIS25), and clove essential oil (OE25) exhibited pronounced radical scavenging activity, achieving DPPH inhibition rates of 86%, 88%, and 84%, respectively. Although these values were slightly lower than that of the standard antioxidant ascorbic acid at 50 μg/mL (AA50:93%), they nonetheless indicate a marked antioxidant capacity. Intermediate concentrations (EU10:82%, BIS10:73%, OE10:79%) retained substantial activity, whereas the lowest concentrations tested (EU5:67%, BIS5:65%, OE5:62%) showed comparatively weaker effects. Statistically, no significant differences were observed among EU25, BIS25, and OE25, nor between EU10 and OE10 or BIS10 and OE10. These results underscore the potential of these compounds as effective radical scavengers, particularly at higher concentrations, and support their relevance as natural antioxidant candidates when compared to ascorbic acid under the same experimental conditions (Figure 2).

**Figure 2:**
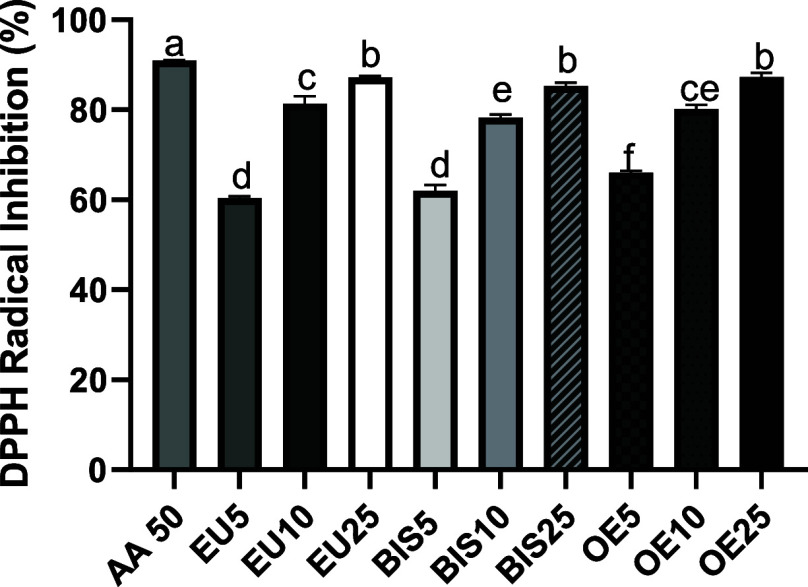
Antioxidant capacity of eugenol, bis-eugenol, and clove essential oil extracts assessed by the DPPH radical scavenging assay (2,2-diphenyl-1-picrylhydrazyl). AA50 refers to the ascorbic acid reference standard (50 μg/mL). EU5, EU10, and EU25 correspond to eugenol at 5, 10, and 25 μg/mL, respectively. BIS5, BIS10, and BIS25 refer to bis-eugenol at 5, 10, and 25 μg/mL, respectively. OE5, OE10, and OE25 represent clove essential oil at 5, 10, and 25 μg/mL, respectively. Data are expressed as mean ± standard deviation. Different lowercase letters (a–f) indicate statistically significant differences between groups (*p* < 0.05, one-way ANOVA followed by Tukey’s post hoc test).

### 3.4 Ferric Reducing Antioxidant Power (FRAP) Assay

The extracts EU25 (285.62 μM) and OE25 (270.79 μM) exhibited the highest FRAP values, demonstrating significant antioxidant capacity. These values were not significantly different from the positive control AA50 (286.00 μM). The extracts EU10 (202.70 μM) and BIS25 (220.46 μM) had intermediate activity and showed no significant difference between them, though both had lower FRAP values than AA50. All other groups, including EU5 (129.77 μM), BIS5 (112.73 μM), BIS10 (133.35 μM), OE5 (129.87 μM), and OE10 (112.27 μM), exhibited significantly lower FRAP values compared to AA50 (*P* < 0.05), as reflected by their distinct statistical groupings in Figure 3.

**Figure 3:**
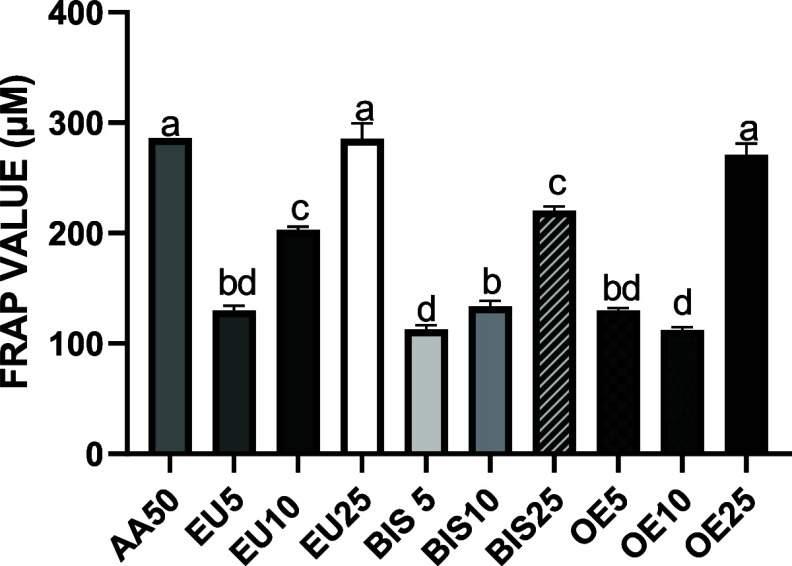
Ferric reducing antioxidant power (FRAP) assay. AA50 refers to the ascorbic acid reference standard (50 μg/mL). EU5, EU10, and EU25 correspond to eugenol at concentrations of 5, 10, and 25 μg/mL, respectively. BIS5, BIS10, and BIS25 refer to bis-eugenol at the same concentrations. OE5, OE10, and OE25 correspond to clove essential oil at concentrations of 5, 10, and 25 μg/mL, respectively. Data are expressed as mean ± standard deviation. Different lowercase letters (a–d) above the bars indicate statistically significant differences between groups (*p* < 0.05, one-way ANOVA followed by Tukey’s post hoc test).

### 3.5 Cell Viability Analysis

In the in vitro analysis, all the extracts exhibited no significant differences for cell viability when compared to the control group (Figure 4). All data represent the mean ± standard deviation of triplicate experiments performed independently three times.

**Figure 4:**
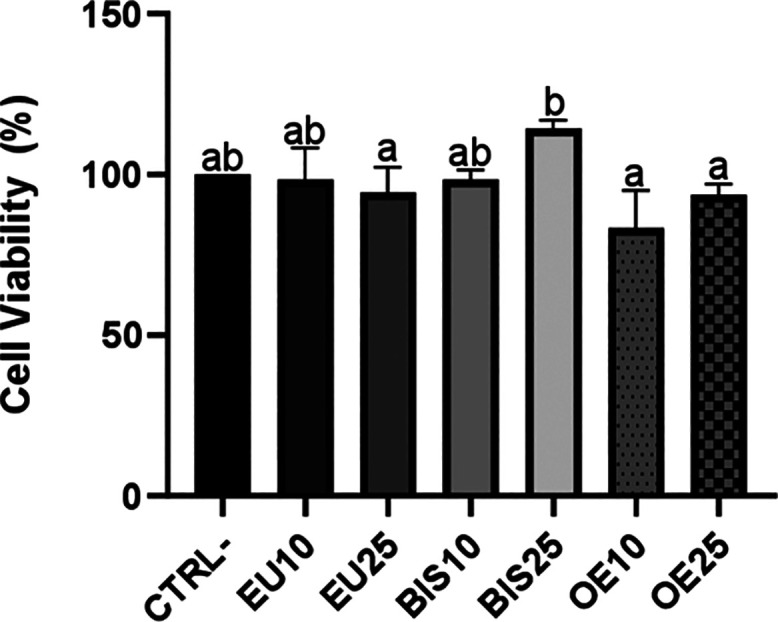
Effect of eugenol, bis-eugenol, and clove essential oil extracts on macrophage cell viability. CTRL– = Negative control group. EU10 and EU25 correspond to eugenol at 10 and 25 μg/mL, respectively (EU10 = 61 μM, EU25 = 152 μM). BIS10 and BIS25 refer to bis-eugenol at 10 and 25 μg/mL, respectively (BIS10 = 30 μM, BIS25 = 76 μM). OE10 and OE25 represent clove essential oil at 10 and 25 μg/mL, respectively (OE10 = 50 μM eugenol equivalent, OE25 = 125 μM eugenol equivalent). Data are expressed as mean ± standard deviation. Different lowercase letters (a, b) above the bars indicate statistically significant differences between groups (*p* < 0.05, one-way ANOVA followed by Tukey’s post hoc test).

### 3.6 Cell Viability Analysis after H_2_O_2_-Induced Oxidative Stress

A decrease in cell viability was observed in the positive control group compared to the negative control. All extracts demonstrated an increase in cell viability relative to the positive control, with BIS25 showing the most pronounced effect. However, no significant difference was observed between the EU and BIS extracts at the concentrations of 10 μg/mL and 25 μg/mL. Additionally, OE10 showed no significant difference compared to BIS10 or EU10 (Figure 5). All data are present as the mean ± standard deviation from three independent experiments, each performed in triplicate.

**Figure 5:**
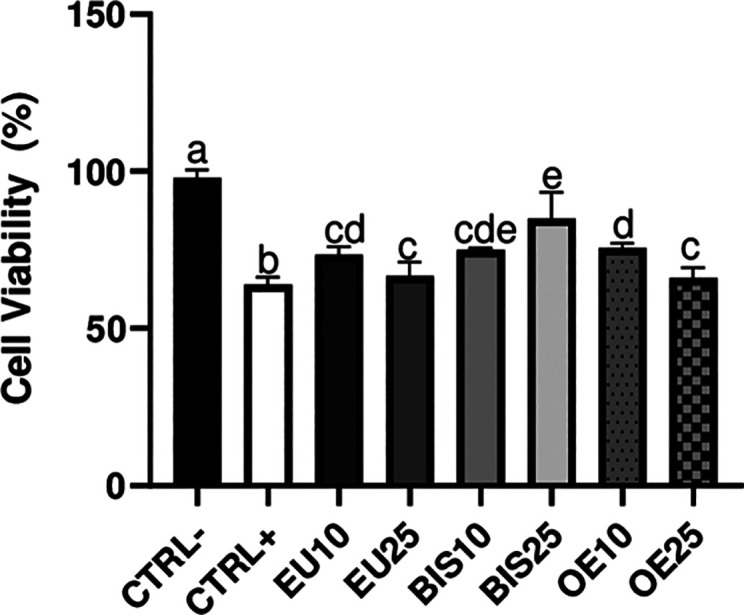
Effect of eugenol, bis-eugenol, and clove essential oil extracts on macrophage cell viability after H_2_O_2_-induced oxidative stress. CTRL– = Negative control group. CTRL+ = Positive control (culture medium +1.5 mM H_2_O_2_). EU10 and EU25 correspond to eugenol at 10 and 25 μg/mL, respectively (EU10 = 61 μM, EU25 = 152 μM). BIS10 and BIS25 refer to bis-eugenol at 10 and 25 μg/mL, respectively (BIS10 = 30 μM, BIS25 = 76 μM). OE10 and OE25 represent clove essential oil at 10 and 25 μg/mL, respectively (OE10 = 50 μM eugenol equivalent, OE25 = 125 μM eugenol equivalent). All extract-treated groups were also exposed to 1.5 mM H_2_O_2_. Data are expressed as mean ± standard deviation. Different lowercase letters (a–e) above the bars indicate statistically significant differences between groups (*p* < 0.05, one-way ANOVA followed by Tukey’s post hoc test).

### 3.7 Evaluation of Antioxidant Enzyme Activity and Oxidative Stress Products

In the analysis of superoxide dismutase (SOD) activity, a significant increase was observed in the positive control group compared to the negative control. Only O25 showed no statistical difference from the positive control among the tested extracts. Additionally, EU25, BIS10, BIS25, OE10, and OE25 did not differ statistically from each other. Furthermore, EU10 was significantly different only from OE25 (Figure 6A).

**Figure 6:**
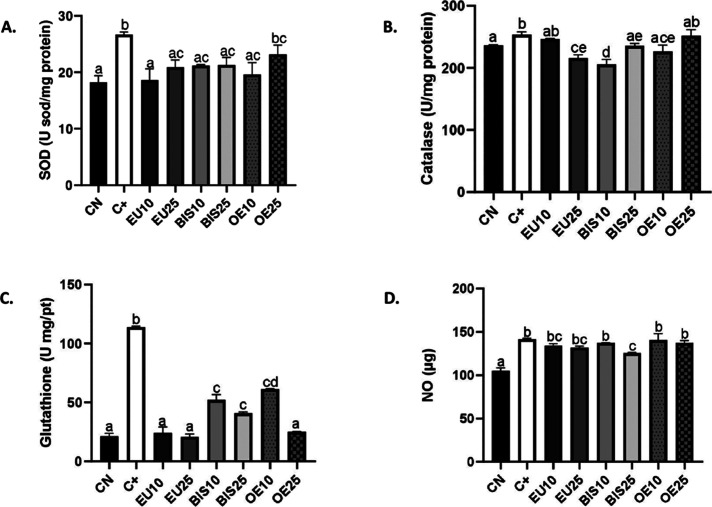
Antioxidant enzyme activity and oxidative stress marker: Catalase (CAT) (A), superoxide dismutase (SOD) (B), glutathione S-transferase (GST) (C), and nitric oxide (NO) (D), a marker of oxidative stress. CN: Negative control group. C+: Positive control (culture medium +1.25 mM H_2_O_2_). EU10 and EU25 correspond to eugenol at 10 and 25 μg/mL, respectively (EU10 = 61 μM, EU25 = 152 μM). BIS10 and BIS25 refer to bis-eugenol at 10 and 25 μg/mL, respectively (BIS10 = 30 μM, BIS25 = 76 μM). OE10 and OE25 represent clove essential oil at 10 and 25 μg/mL, respectively (OE10 = 50 μM eugenol equivalent, OE25 = 125 μM eugenol equivalent). All extract-treated groups were also exposed to 1.25 mM H_2_O_2_. Data are presented as mean ± standard deviation (SD). Different lowercase letters (a–e) above the bars indicate statistically significant differences between groups (*p* < 0.05), as determined by one-way ANOVA followed by Tukey’s post hoc test. Groups sharing the same letter are not significantly different from each other.

In the catalase activity analysis, a significant increase was observed in the positive control group compared to the negative control. Among the tested extracts, only OE25 exhibited values statistically similar to those of the positive control. The greatest reduction in catalase activity was observed in the EU25 and BIS10 groups, which were significantly different from all other extracts (Figure 6B).

In the analysis of glutathione–S-transferase activity, a significant increase was observed in the positive control group compared to the negative control. All tested extracts significantly reduced glutathione-S-transferase activity levels compared to the positive control, with extracts EU10, EU25, and OE25 showing the most pronounced inhibitory effects (Figure 6C).

A significant increase in nitric oxide production was observed in the positive control group compared to the negative control. Among the tested extracts, only BIS25 significantly reduced nitric oxide production relative to the positive control and exhibited no significant difference from those of the EU10 and EU25 groups. The other extracts did not differ statistically from each other (Figure 6D).

### 3.8 mRNA Extraction and Quantitative Real-Time qPCR

Molecular analysis revealed a significant upregulation of TLR-4 expression in the positive control compared to the negative control. In contrast, all experimental groups exhibited a reduction in TLR-4 expression compared to the positive control. Notably, EU10, BIS25, and OE25 demonstrated the most substantial reduction of TLR-4 expression, with levels falling below those of the negative control (Figure 7).

**Figure 7:**
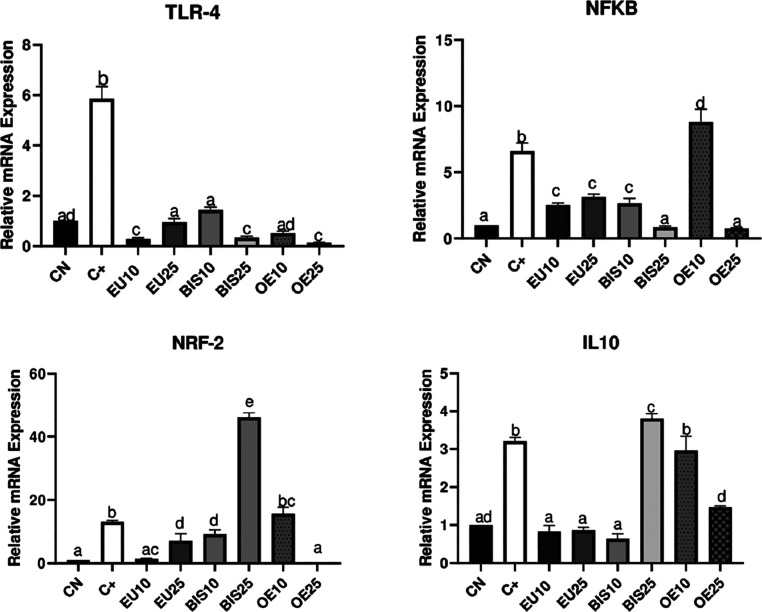
Relative mRNA expression of inflammatory and anti-inflammatory markers in different experimental conditions. The expression levels of IL-10, TLR, NRF2, and NRKB were assessed by qPCR in the following groups: CN (negative control), C+ (positive control, LPS-stimulated cells). EU10 and EU25 correspond to eugenol at 10 and 25 μg/mL, respectively (EU10 = 61 μM, EU25 = 152 μM). BIS10 and BIS25 refer to bis-eugenol at 10 and 25 μg/mL, respectively (BIS10 = 30 μM, BIS25 = 76 μM). OE10 and OE25 represent clove essential oil at 10 and 25 μg/mL, respectively (OE10 = 50 μM eugenol equivalent, OE25 = 125 μM eugenol equivalent). All extract-treated groups were coexposed to LPS. Data are presented as mean ± standard deviation (SD). Different lowercase letters (a–d) above the bars indicate statistically significant differences between groups (*p* < 0.05), as determined by one-way ANOVA followed by Tukey’s post hoc test. Groups sharing the same letter are not significantly different from each other.

Analysis of NF-κB expression revealed a significant upregulation in the positive control compared to the negative control. While most of extracts reduced NF-κB expression, OE notably induced its expression. Furthermore, BIS25 and OE25 exhibited the most substantial downregulation, significantly exceeding the inhibitory effect observed with the other tested extracts.

NRF-2 expression was significantly higher in the positive control compared to the negative control. While OE10 maintained NFR-2 expression levels comparable to the positive control, EU10, EU25, and BIS10 exhibited a significant downregulation of NFR-2 expression relative to the positive control, with no statistically significant difference between these two groups. Notably, BIS25 demonstrated the highest NRF-2 expression, exceeding both the positive control and all other experimental extracts.

IL-10 expression analysis revealed significant upregulation in the positive control compared to the negative control (*p* > 0.05). BIS25 increased IL-10 levels, while OE10 maintained them relative to positive control. All other treatment groups exhibited significant downregulation (*p* < 0.05). Notably, EU10, EU25, and BIS10 exhibited the most pronounced inhibitory effects on IL-10 expression, with no significant difference among them.

### 3.9 TNF-α Quantification Using the ELISA Kit

The positive control group exhibited an increase in TNF-α concentration compared to the negative control. Neither EU10 nor EU25 resulted in a reduction of TNF levels compared to the positive control. In contrast, all concentrations of bis-eugenol and clove essential oil significantly downregulated TNF-α levels, with no significant differences observed between the different concentrations (Figure 8).

**Figure 8:**
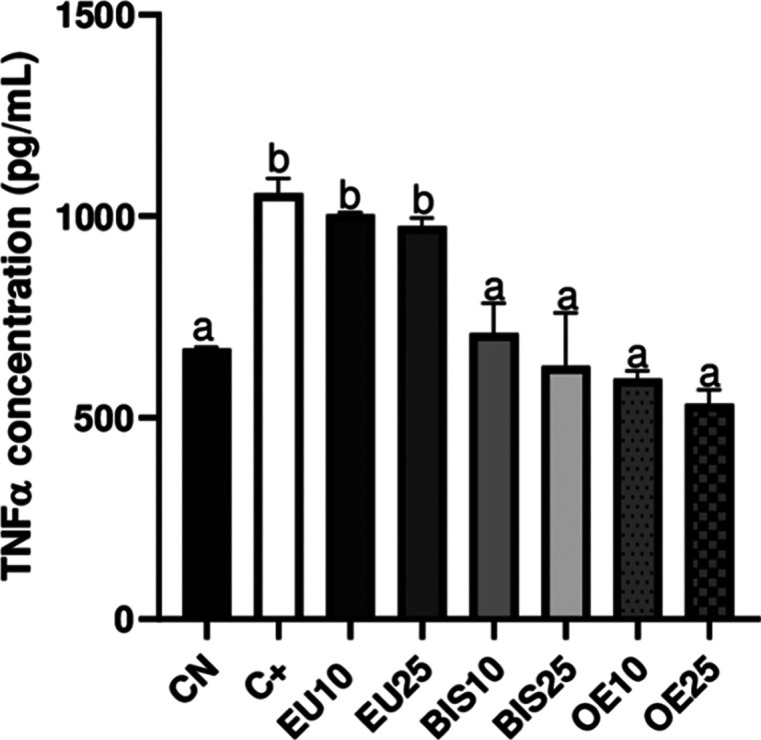
CN = Negative control group. CTRL+ = Positive control group (culture medium with LPS (10 μg/mL) added). EU10 and EU25 correspond to eugenol at 10 and 25 μg/mL, respectively (EU10 = 61 μM, EU25 = 152 μM). BIS10 and BIS25 refer to bis-eugenol at 10 and 25 μg/mL, respectively (BIS10 = 30 μM, BIS25 = 76 μM). OE10 and OE25 represent clove essential oil at 10 and 25 μg/mL, respectively (OE10 = 50 μM eugenol equivalent, OE25 = 125 μM eugenol equivalent). All extract-treated groups were also exposed to LPS. Data are expressed as mean ± standard deviation. Different lowercase letters (a, b) above the bars indicate statistically significant differences between groups (*p* < 0.05), as determined by one-way ANOVA followed by Tukey’s post hoc test. Groups sharing the same letter are not significantly different from each other.

## 4 Discussion

This study is the first to compare the effects of eugenol, bis-eugenol, and clove essential oil on oxidative stress and inflammation process control. Given the well-established antioxidant and anti-inflammatory properties of eugenol, this study aimed to evaluate whether its derivative forms, bis-eugenol and clove essential oil, exhibit distinct modulatory effects on these biological processes. The clove essential oil contains a complex mixture of bioactive compounds, and this mix may influence its antioxidant and anti-inflammatory activity differently. Thus, our investigation seeks to clarify the differences between these three forms and their impact on the cellular response to oxidative and inflammatory stress. The GC-MS results are consistent with the values reported in the literature, which indicate that clove-derived EOs normally contain between 45% and 90% eugenol. Quantification by ^1^H NMR showed a value similar to that obtained by GC-MS, reinforcing the reliability of the analytical methods employed. COSY analysis showed that the chemical structure of eugenol was preserved in the EO. The presence of the band at 1768 cm^‑1^ in the IR spectrum confirms the existence of eugenol acetate (Figure S7, Supporting Information), differentiating it from pure eugenol. These findings indicate that the extracted essential oil has a chemical profile compatible with other samples described in the literature. Phenols (such as eugenol) and phenyl ethers undergo oxidative coupling in the presence of Fe (III) salts, leading to the formation of new carbon–carbon bonds. Bis eugenol was obtained as a yellowish solid with a 97% yield. It was characterized by nuclear magnetic resonance and infrared spectroscopy. The spectroscopic data are in agreement with those reported in the literature.^27^

One of the most widely used methods for assessing antioxidant capacity is the DPPH radical scavenging assay. In our studies, the highest concentration of the three extracts tested (25 μg/mL) showed outstanding performance (80% inhibition), suggesting the presence of bioactive molecules with high efficiency in neutralizing free radicals, presenting antioxidant activity similar to that of the ascorbic acid used as the reference standard. In the Ferric Reducing Antioxidant Power (FRAP) test, we observed a similar pattern, in which the highest concentrations of eugenol, clove essential oil, and bis-eugenol (25 μg/mL) exhibited the greatest ferric ion reducing activity, corroborating the results obtained in DPPH radical inhibition. Gülçin et al. (2011) demonstrated that eugenol has a more potent antioxidant and radical scavenging activity compared to widely used standard antioxidants such as Trolox, butylated hydroxytoluene, butylated hydroxytoluene and α-tocopherol.^28^ These findings are consistent with our results since, in addition to pure eugenol, both bis-eugenol and the clove essential oil have significant concentrations of eugenol, which justify their high antioxidant capacity. The antioxidant activity of eugenol seems to be related to its ability to donate hydrogen atoms, which contributes to the neutralization of free radicals.^29^ This property is influenced by hydroxyl groups in the phenolic ring, which facilitate electron donation and help interrupt oxidative chain reactions.^29,30^ However, in our analysis, only the highest concentrations of eugenol, clove essential oil, and bis eugenol showed antioxidant activity comparable to ascorbic acid, suggesting that its effectiveness depends on the dose used. Although bis-eugenol and eugenol differ in molecular structure and weight, their comparable antioxidant effects, particularly at higher concentrations, suggest that these differences did not substantially impact their efficacy under our experimental conditions. Nonetheless, future studies could explore antioxidant effects based on molar equivalents or redox reactivity to further elucidate mechanistic distinctions between these compounds. After investigating the antioxidant effects of the extracts and their dose-dependent behavior, two concentrations were selected for further in vitro analysis: 10 μg/mL and 25 μg/mL. Our findings demonstrate that all concentrations of the different samples did not adversely affect cell viability, suggesting that the extracts do not exhibit citotoxicity. According to the literature, eugenol has been reported to be toxic at high concentrations, where it can cause protein inactivation and interact with DNA, resulting in cellular damage.^31,32^ However, the concentrations used in this study are within safe levels, and no significant adverse effects were observed in the cells under investigation. The observed recovery in cell viability following H_2_O_2_-induced oxidative stress suggests that the tested compounds exert protective effects likely mediated by multiple antioxidant mechanisms. Phenolic compounds such as eugenol and bis-eugenol are known to act through both direct and indirect antioxidant pathways. Directly, they can scavenge reactive oxygen species (ROS) due to their redox-active hydroxyl groups, which stabilize free radicals by donating hydrogen atoms.^12^ Indirectly, they may modulate intracellular signaling pathways, leading to the upregulation of endogenous antioxidant defenses, such as glutathione, superoxide dismutase, and catalase. Moreover, these compounds may help preserve membrane integrity and reduce oxidative damage to key biomolecules, including lipids, proteins, and DNA.^33^ The more pronounced protective effect observed with bis-eugenol at 25 μg/mL may be attributed to its dimeric structure, which provides additional phenolic sites for ROS neutralization and enhanced radical stabilization via resonance.

Studies show that eugenol has antioxidant properties; however, it can also lead to the formation of phenoxyl radicals (oxygen-centered free radicals), which can have a catalytic impact, promoting the formation of more free radicals.^12^ The formation of these radicals depends on the energy required to remove a hydrogen from the OH group of eugenol. According to the aforementioned study, this energy (Δ*H*) is lower for eugenol than for bis-eugenol, indicating that eugenol forms radicals more easily.^34^ We hypothesized that both eugenol and bis-eugenol exerted a protective effect against H_2_O_2_-induced damage due to their antioxidant properties. However, as bis-eugenol generates fewer phenoxyl radicals, its protection may have been more stable and effective. Despite the promising results, this study is limited by the absence of detailed analyses of the molecular structure and chemical stability of the compounds, such as computational modeling (QSAR or molecular docking), which could provide additional support to explain the greater activity of bis-eugenol. Without these analyses, the attribution of bis-eugenol’s superiority to its structure and chemical stability remains a hypothesis that should be confirmed in future studies.

Upon exposure to hydrogen peroxide (H_2_O_2_), cells experience an increased generation of reactive oxygen species (ROS), including superoxide anion (O_2_-). In biological systems, H_2_O_2_ can be produced endogenously through the dismutation of O_2_-, a reaction catalyzed by the enzyme superoxide dismutase (SOD).^35^ Under conditions of intense oxidative stress, such as those observed in the positive control treated with H_2_O_2_, the activity of antioxidant enzymes such as SOD, catalase (CAT) and glutathione S-transferase (GST), is upregulated. These enzymes play an essential role in mitigating oxidative damage by neutralizing free radicals and restoring cellular homeostasis.^36^ In our study, the activity levels of these enzymes were lower in the groups treated with the extracts compared to the positive control. This reduction does not indicate enzyme inhibition, but rather suggests a decreased oxidative burden, which, in turn, reduced the requirement for activation of the endogenous antioxidant defense system. In this context, the extracts, particularly eugenol and bis-eugenol, likely acted as direct antioxidants, scavenging ROS and preventing their accumulation before the enzymatic defenses needed to be substantially activated. Specifically regarding GST, which is involved in the detoxification of electrophilic compounds and products of lipid peroxidation, the reduced activity observed in treated groups indicates a lower generation of such reactive byproducts.^37^ Consistent with our findings, previous studies have reported that exposure to eugenol can lead to a gradual decrease in GST activity, suggesting that this compound may modulate or downregulate GST expression or activity depending on the cellular context.^38,39^ These observations support the hypothesis that the extracts conferred early antioxidant protection, thereby reducing the downstream metabolic demand for GST-mediated conjugation and detoxification pathways. In this context, the extracts may have contributed to maintaining cellular homeostasis by preventing the excessive accumulation of H_2_O_2_ and limiting the activation of secondary antioxidant mechanisms. Moreover, in contrast to antioxidant enzymes, nitric oxide (NO) production remained largely unchanged in response to most extracts, with the exception of BIS25, which significantly reduced this marker. This finding suggests that while the extracts primarily function by preventing the excessive ROS formation, the modulation of the NO pathway appears to be a specific effect of BIS25. This effect may be associated with a direct interaction with nitric oxide synthase (NOS) or the regulation of inflammatory signaling that modulates NO production.

Furthermore, regarding inflammatory markers, all the extract-treated samples showed a reduction in TLR4 levels compared to the C+, suggesting that the compounds tested were able to downregulate this pathway, possibly attenuating the inflammatory response. Analysis of NF-κB activation corroborated the TLR4 analyses, despite revealing distinct behaviors among the samples. It is important to highlight that TLR4-NF-κB is one of the most important pathways that should be downregulated to control the OxInflammation process. This pathway has a central role in the control of the multiple genes pro-inflammatory, inflammasome activated, respiratory burst and in the development of a pro-oxidative microenvironment.^40^ Although most of the treatments with Eugenols resulted in a decrease in NF-κB levels, clove essential oil (10 uM) showed an increase in the activation of this pathway, even surpassing the levels of positive control. Furthermore, the present study revealed that the expression of Nrf2 and IL10 was increased by treatment with bis eugenol (25 uM), following the results presented by the TLR4-NF-κB pathway. We can see that the inflammatory response induced by lipopolysaccharide (LPS) is closely associated with the activation of Toll-like receptor 4 (TLR4) and the subsequent activation of the nuclear factor kappa B (NF-κB) pathway in the positive group. The interaction between TLR4 and NF-κB in the treatments evaluated strengthens the hypothesis that modulation of these pathways is directly related to the anti-inflammatory effect of the compounds. When LPS binds to the Toll-like receptor (TLR) 4, it triggers intracellular signaling, leading to the activation and translocation of the nuclear transcription factor NF-κB into the nucleus.^41^ However, neither TLR4 activation nor NF-κB translocation to the nucleus were observed in the BIS25 treatment, as well as in the EUG10, EUG25, BIS10, and OE25 groups. This may explain the reduction in the inflammatory response in these groups, with BIS25 showing the most pronounced effect.

Previous studies indicate that anti-inflammatory compounds suppress the NF-κB signaling pathway and activate the Nrf2 signaling pathway.^42^ In the case of BIS25, the increase in nuclear localization of NF-κB, along with the reduction in nuclear localization of Nrf2, which typically occurs following LPS treatment, was reversed. Moreover, it is already know that there is a direct relationship between the increase in ROS levels and NF-κB activation, triggering inflammation.^43^ As demonstrated previously in our study, BIS25 prevented excessive ROS formation, further supporting its ability to simultaneously activate endogenous antioxidant defenses and inhibit pro-inflammatory pathways. These results suggest that BIS25 may effectively modulate the balance between inflammatory and oxidant processes, thereby promoting cellular protection. The significant increase in IL-10 observed only in the BIS25-treated group suggests that this extract plays a crucial role in inflammation resolution. IL-10 is a key anti-inflammatory cytokine known for attenuating the inflammatory response following pathogen invasion and protecting the host from excessive inflammation.^44^ Its upregulation in response to BIS25 treatment indicates that this extract promotes an adaptive cellular response, modulating inflammatory and oxidative stress processes. This effect may explain its superior performance in cytoprotection, cell proliferation, and oxidative stress assays. The significant increase in IL-10 observed only in the BIS25-treated group may be related to its unique ability to activate the NRF2 pathway more effectively than the other treatments. While EU10, EU25, and BIS10 did not significantly increase NRF2 expression, BIS25 induced a notable upregulation of this transcription factor, which is known to enhance antioxidant defenses and modulate anti-inflammatory cytokines such as IL-10.^45^ This differential activation of NRF2 likely accounts for the absence of increased IL-10 expression in the other groups, highlighting the superior capacity of bis-eugenol at 25 μg/mL to promote an adaptive cellular response and exhibit significant therapeutic potential for treating inflammatory and degenerative conditions associated with oxidative stress.

Regarding clove essential oil, OE 25 also demonstrated a strong anti-inflammatory effect, significantly reducing the expression of TLR4 and NF-κB but without a concomitant increase in NRF2 and IL-10. However, for OE 10, an increase in NF-κB expression was observed, suggesting that this compound may activate additional pathways that are not directly dependent on TLR4, which merits further investigation.Our results indicate that the effects of clove essential oil are dose-dependent and influenced by the chemical complexity of the extract. Notably, OE10 reduced oxidative stress markers while simultaneously increasing NF-κB expression, suggesting persistent activation of inflammatory signaling at this concentration. This effect may not be solely attributable to eugenol, as clove essential oil contains other active constituents such as β-caryophyllene and eugenyl acetate, which may modulate NF-κB through alternative or additive mechanisms. In contrast, higher concentrations (OE25) suppressed TLR4/NF-κB signaling, albeit without a proportional upregulation of antioxidant markers such as NRF2. These findings support a biphasic response model, wherein low concentrations may preferentially activate antioxidant pathways but are insufficient to fully suppress inflammation, whereas higher concentrations exert a broader inhibitory effect on inflammatory signaling but may limit compensatory antioxidant responses.

Similarly, pure eugenol positively affected enzymatic activity, suggesting a positive impact on oxidative stress regulation. Additionally, it significantly reduced the expression of NF-κB and TLR4, reinforcing its anti-inflammatory potential. However, unlike BIS25, EUG10, EUG25, and BIS10 did not induce an increase in IL-10 or NRF2 expression, suggesting that their mechanism of action may rely primarily on the direct inhibition of inflammatory signaling rather than on the activation of adaptive antioxidant and anti-inflammatory pathways.These findings indicate that, while eugenol (10, 25) and BIS10 is effective in downregulating key inflammatory mediators, its ability to stimulate endogenous protective responses appears to be limited compared to BIS25. This distinction highlights the potential advantage of bis-eugenol in providing a more comprehensive modulation of inflammation and oxidative stress, possibly through NRF2 activation and IL-10 upregulation. One of the primary mechanisms by which IL-10 exerts its anti-inflammatory effect is through the downregulation of pro-inflammatory genes, such as those encoding tumor necrosis factor alpha (TNF-α).^46^ However, our results show that eugenol alone did not upregulate IL-10 expression or reduce TNF-α expression, suggesting that this specific inflammatory modulation pathway was not activated. On the other hand, eugenol also did not induce a significant increase in pro-inflammatory cytokines, indicating that its effect was not clearly inflammatory nor mediated by classical anti-inflammatory pathways, such as IL-10 activation. However, considering that eugenol effectively reduced the expression of TLR4 and NF-κB, its anti-inflammatory activity may be more related to the direct inhibition of these factors than to the activation of regulatory mechanisms. Thus, our findings suggest that eugenol exerts its anti-inflammatory effects predominantly through modulating the TLR4/NF-κB signaling pathway, without triggering a compensatory IL-10-mediated response. It is noteworthy, however, that the absence of protein-level validation limits the robustness of these mechanistic insights and underscores the need for further confirmatory studies.

## 5 Conclusions

This study represents the first comparative analysis of the effects of eugenol, bis-eugenol, and clove essential oil on oxidative stress and inflammation. Our findings indicate that, among the tested compounds, bis-eugenol at 25 μg/mL exhibited the most pronounced antioxidant and anti-inflammatory effects. Its distinct chemical structure appears to confer enhanced stability and reduced phenoxy radical generation, which may explain its superior efficacy in cellular protection and inflammatory modulation. Notably, bis-eugenol was the only compound that simultaneously suppressed TLR4/NF-κB pathways while upregulating NRF2 and IL-10, suggesting that its mechanism of action involves both direct inflammatory inhibition and the activation of endogenous protective pathways. The mRNA expression was confirmed by the TNF-α quantification, cell viability, and antioxidant enzyme analyses. Clove essential oil exhibited a biphasic response, with lower concentrations promoting the antioxidant response and reducing ROS levels, while higher concentrations suppressed inflammation via inhibition of the TLR4/NF-κB pathway but without a corresponding enhancement of antioxidant defenses. In contrast, pure eugenol effectively reduced NF-κB and TLR4 expression. However, it failed to increase NRF2 and IL-10, indicating that its anti-inflammatory effects are primarily mediated through direct pathway modulation rather than broader adaptive responses. Thus, we suggest that bis-eugenol is the most promising candidate among the compounds evaluated due to its more stable and efficient antioxidant and anti-inflammatory properties. Nevertheless, further investigations are needed to elucidate the influence of its chemical structure on these molecular mechanisms and to substantiate its primary role in mediating these effects. Such studies will enhance our comprehension of bis-eugenol therapeutic potential and inform its prospective clinical applications.

## Supplementary Material



## Data Availability

All acquired data are systematically presented within the text.

## Notes

Additional experimental details, characterization data of bis-eugenol, NMR and IR spectra, GC-MS chromatograms of clove essential oil, and supplementary figures related to eugenol quantification (PDF)
