# Supplementary material for: Antioxidant and Anti-inflammatory Activity of Eugenol, Bis-eugenol, and Clove Essential Oil: An In Vitro Study
Source: ACS Omega. 2025 Jul 10;10(28):31033–45. doi: 10.1021/acsomega.5c04146 (PMC12290672; doi:10.1021/acsomega.5c04146)
Supplement: Supplementary file 1 [file ao5c04146_si_001.pdf]

Antioxidant and Anti-inflammatory Activity of Eugenol, Bis-eugenol, and Clove Essential Oil: An In Vitro Study

Eduarda Pires Costa[1], Manoela Maciel[2], Rosinéa Aparecida de Paula[2], Danilo Aniceto da Silva[3], Renata Pereira Lopes[3], Robson Ricardo Teixeira[3], Reggiani Vilela Gonçalves*[2,4]

[1] Department of General Biology, Federal University of Viçosa, Avenida PH Rolfs, s/n, Campus Universitário, Viçosa, MG, 36570-900, Brazil; [2] Department of Animal Biology, Federal University of Viçosa, Avenida PH Rolfs, s/n, Campus Universitário, Viçosa, MG, 36570-900, Brazil; [3] Department of Chemistry, Federal University of Viçosa, Avenida PH Rolfs, s/n, Campus Universitário, Viçosa, MG, 36570-900, Brazil; [4] Plants for Human Health Institute, Animal Science, North Carolina State University, 500 Laureate Way, Kannapolis, NC 28081, USA

* Corresponding author: Reggiani Vilela Gonçalves; rvilela@ncsu.edu / reggiani.goncalves@ufv.br .

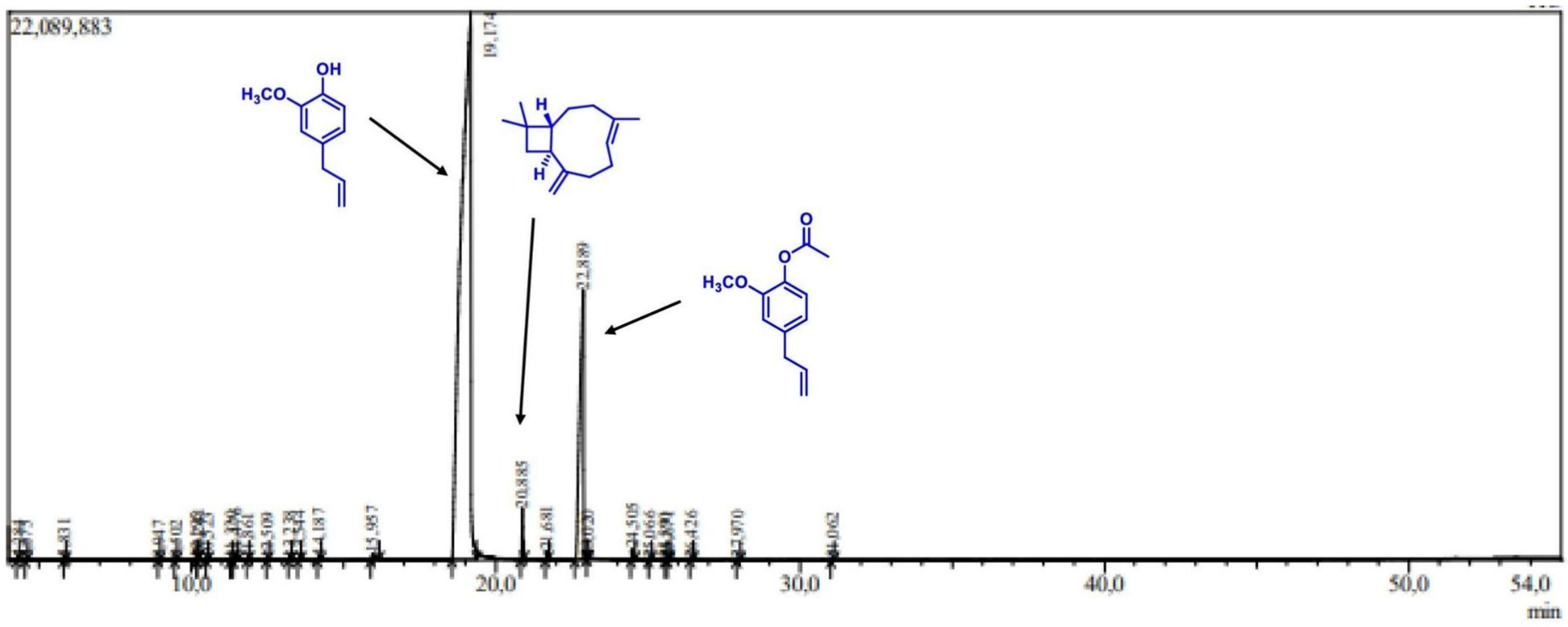

Figure S1. Clove essential oil chromatogram obtained by GC-MS analysis**.** The structures of the main components are shown.

Table S1. Results of the CG-MS analysis of clove essential oil.

Component	Structure	Retention Time (min.)	Percentage (%)[a]
Eugenol	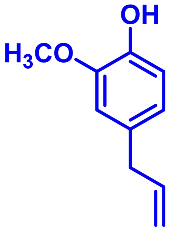	19.2	82.2
β-Caryophyllene	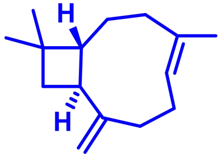	20.0	1
Eugenol acetate	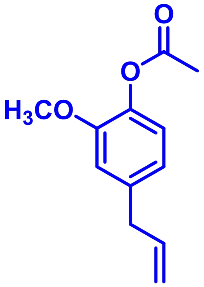	22.9	15.8

[a]Determined by CG-MS analysis as described in the Material and Methods section.

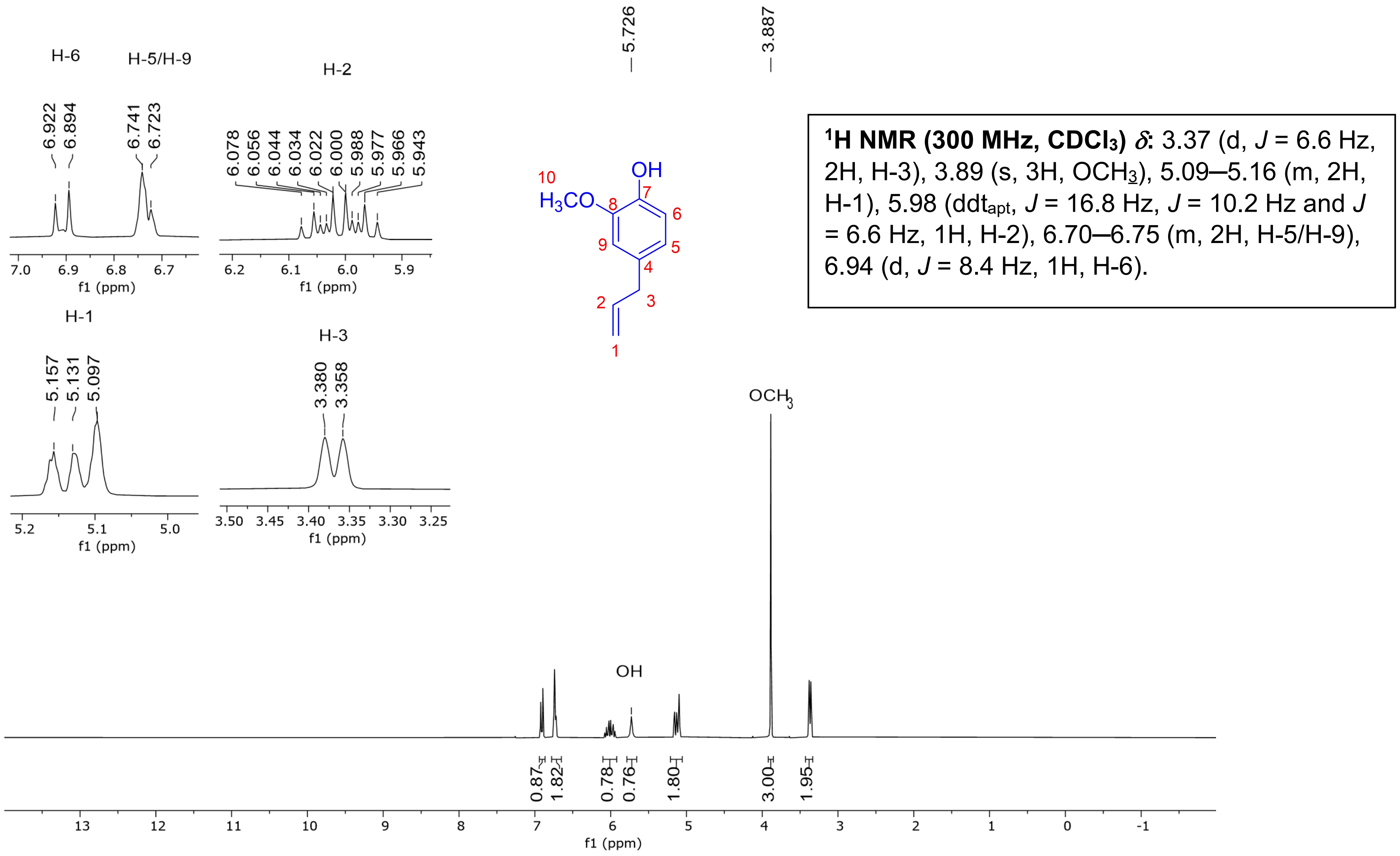

^{1}H NMR (300 MHz, $CDCl_3$) δ: 3.37 (d, *J* = 6.6 Hz, 2H, H-3), 3.89 (s, 3H, OCH_3), 5.09–5.16 (m, 2H, H-1), 5.98 (ddt_{apt}, *J* = 16.8 Hz, *J* = 10.2 Hz and *J* = 6.6 Hz, 1H, H-2), 6.70–6.75 (m, 2H, H-5/H-9), 6.94 (d, *J* = 8.4 Hz, 1H, H-6).

Figure S2. ^{1}H NMR ($CDCl_3$, 300 MHz) of pure eugenol.

A

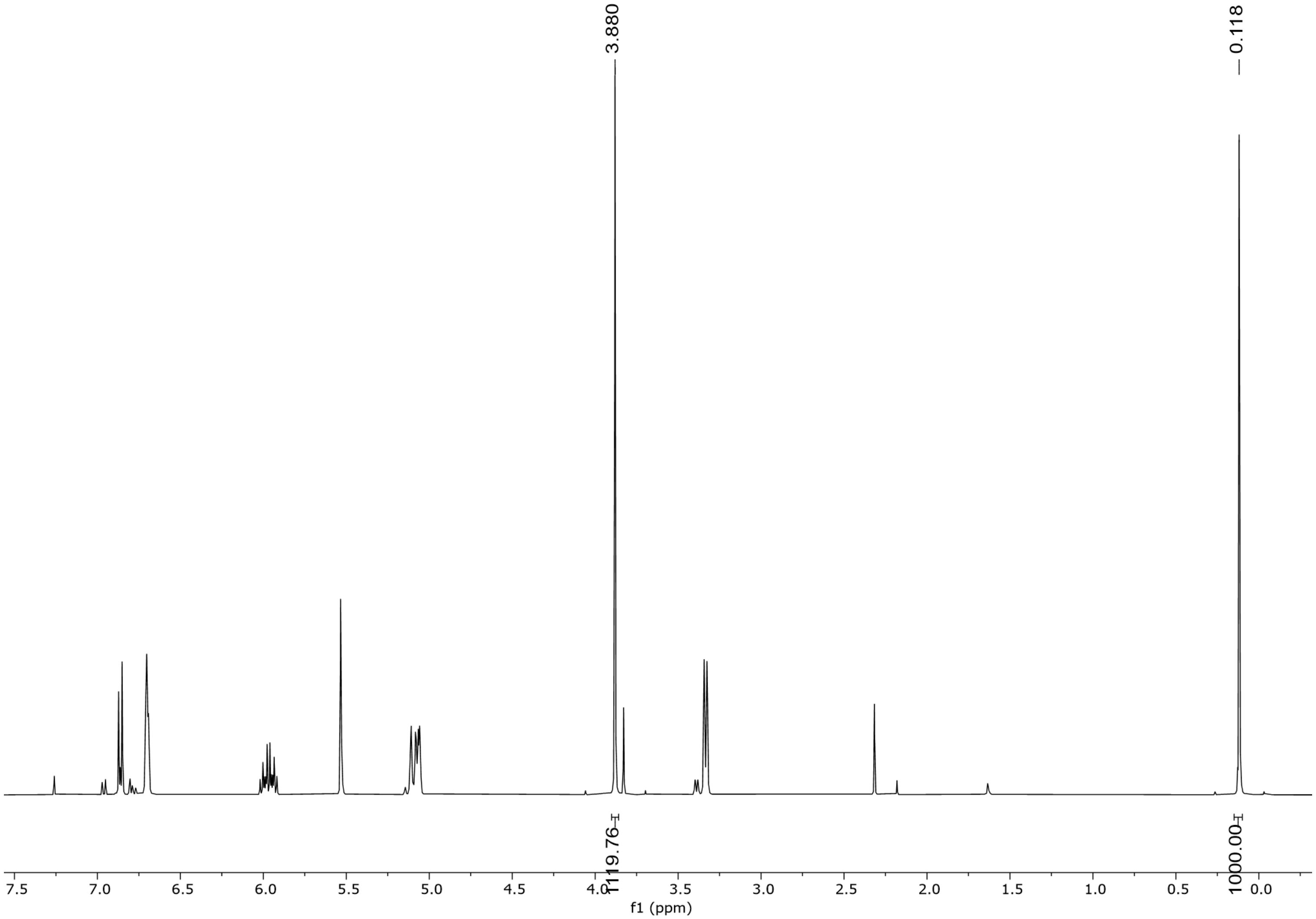

Percentage of eugenol in the clove EO determined as described in the Materials and Methods: 86.85%

B

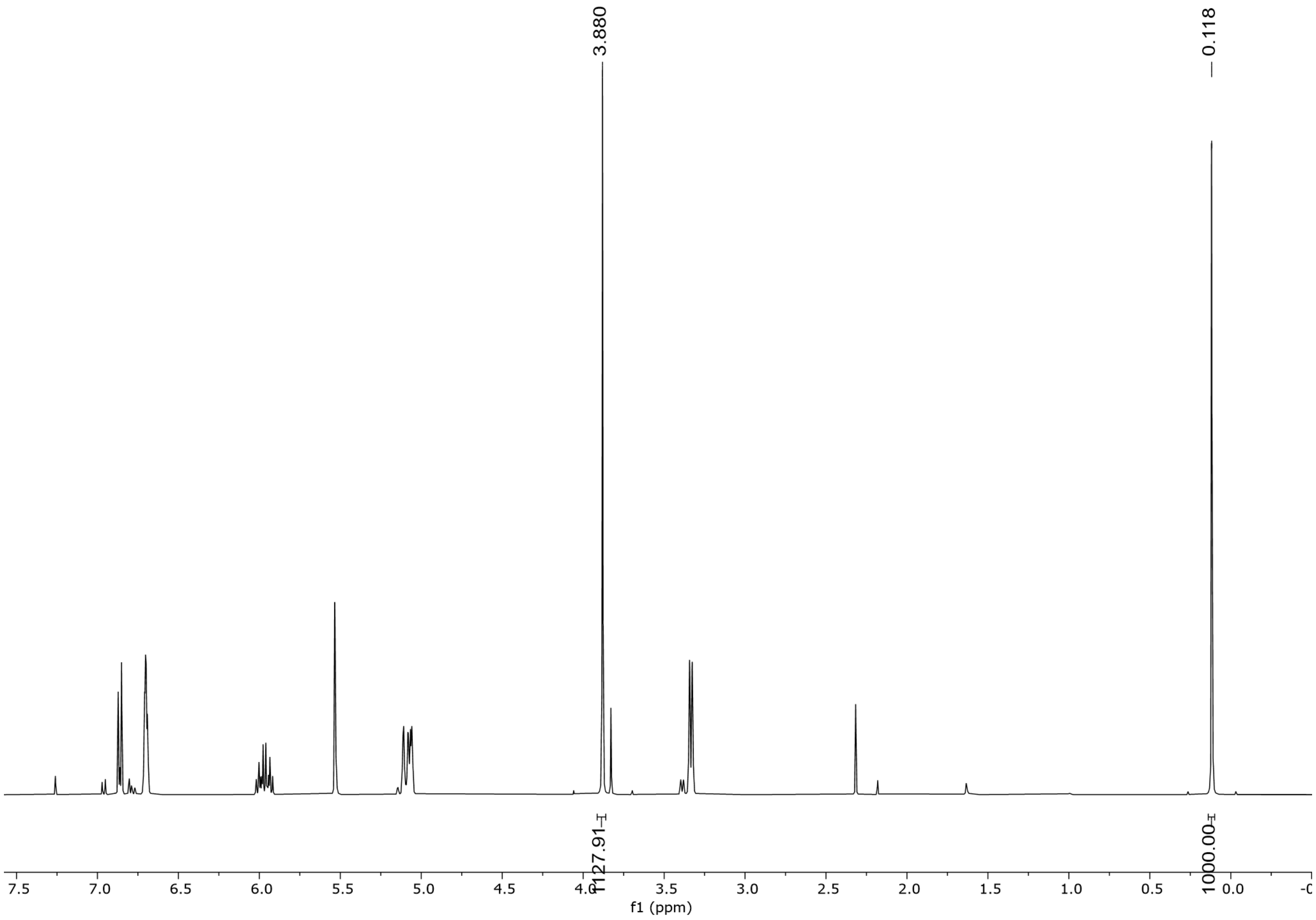

Percentage of eugenol in the clove EO determined as described in the Materials and Methods: 86.24%

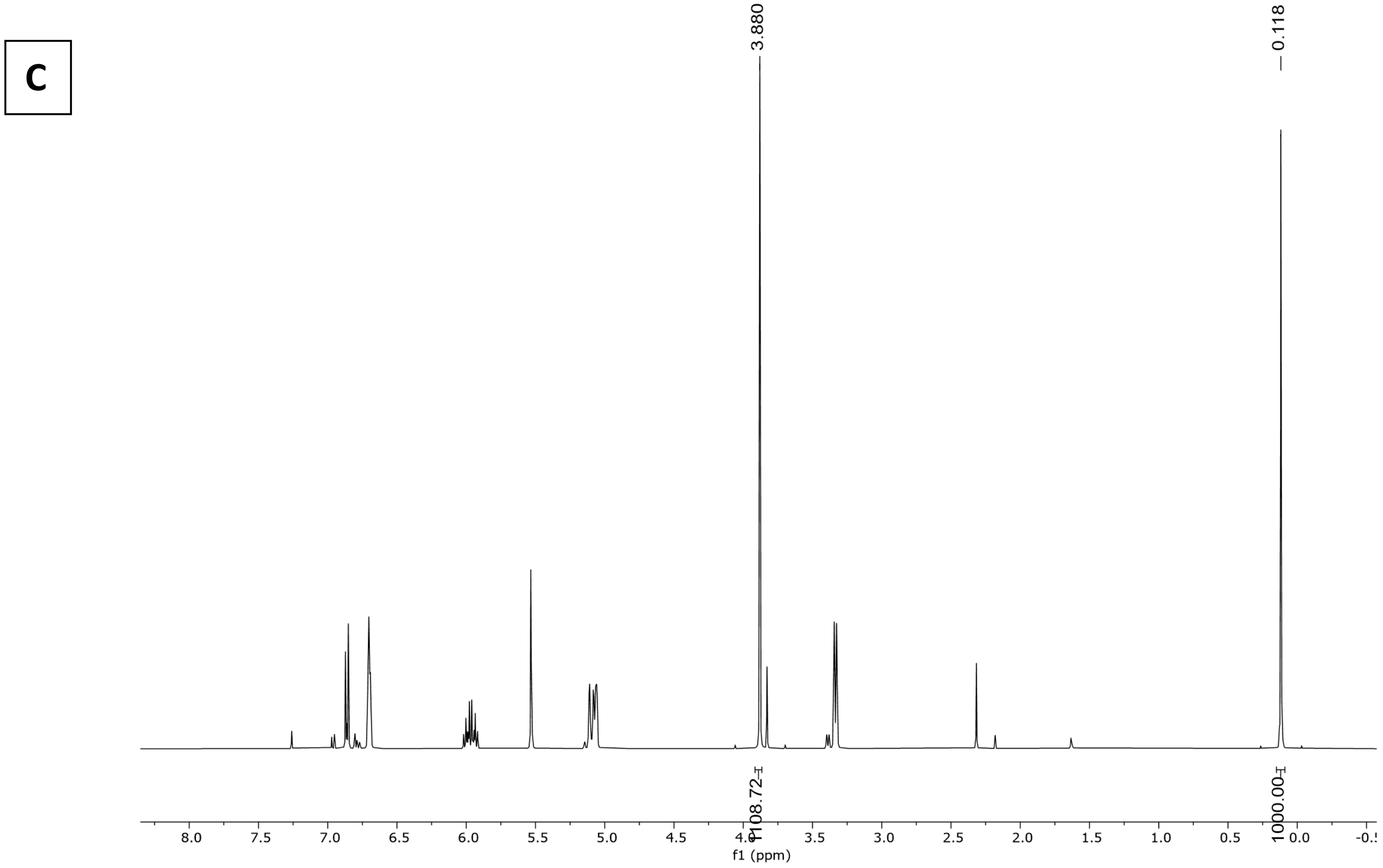

Percentage of eugenol in the clove EO determined as described in the Materials and Methods: 86.75%.

Figure S3. ^{1}H NMR (400 MHz, $CDCl_3$) spectra of clove EO. Three acquisitions (A, B, and C) were made for the quantification of eugenol in the EO. Average eugenol content = 86.6 ± 0.27 %

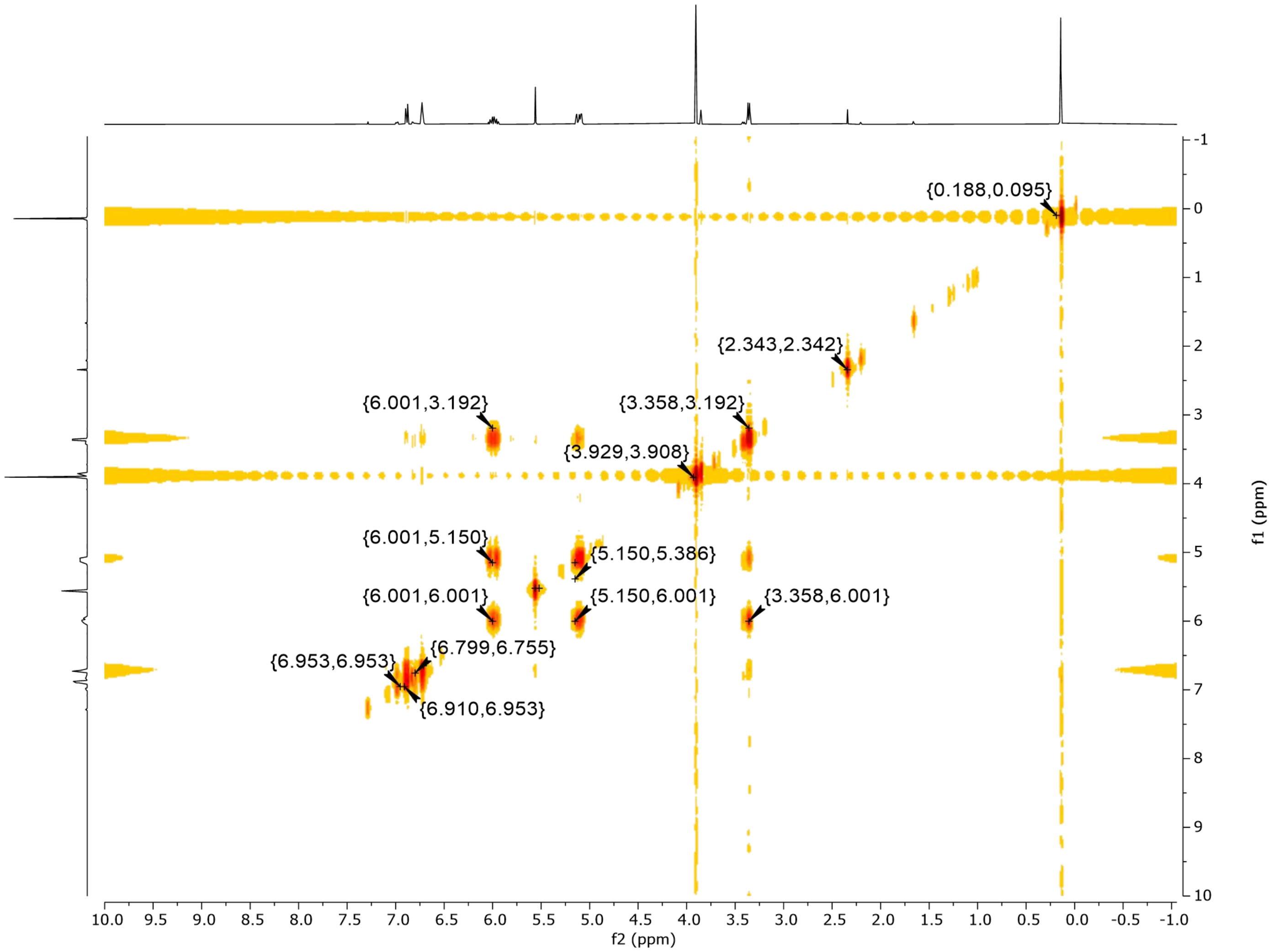

Figure S4. COSY contour plot of EO.

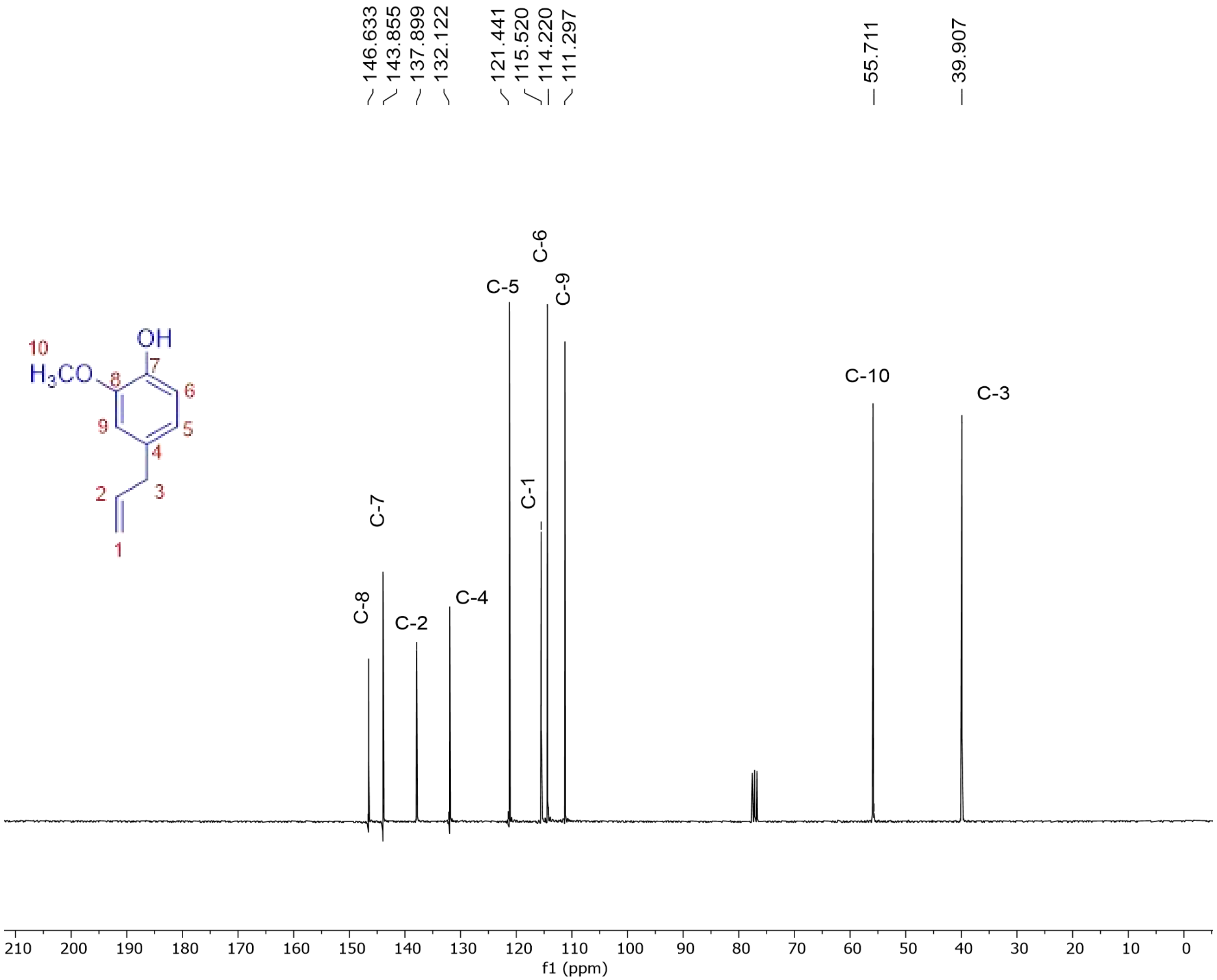

Figure S5. ^{13}C NMR (75 MHz, $CDCl_3$) of pure eugenol.

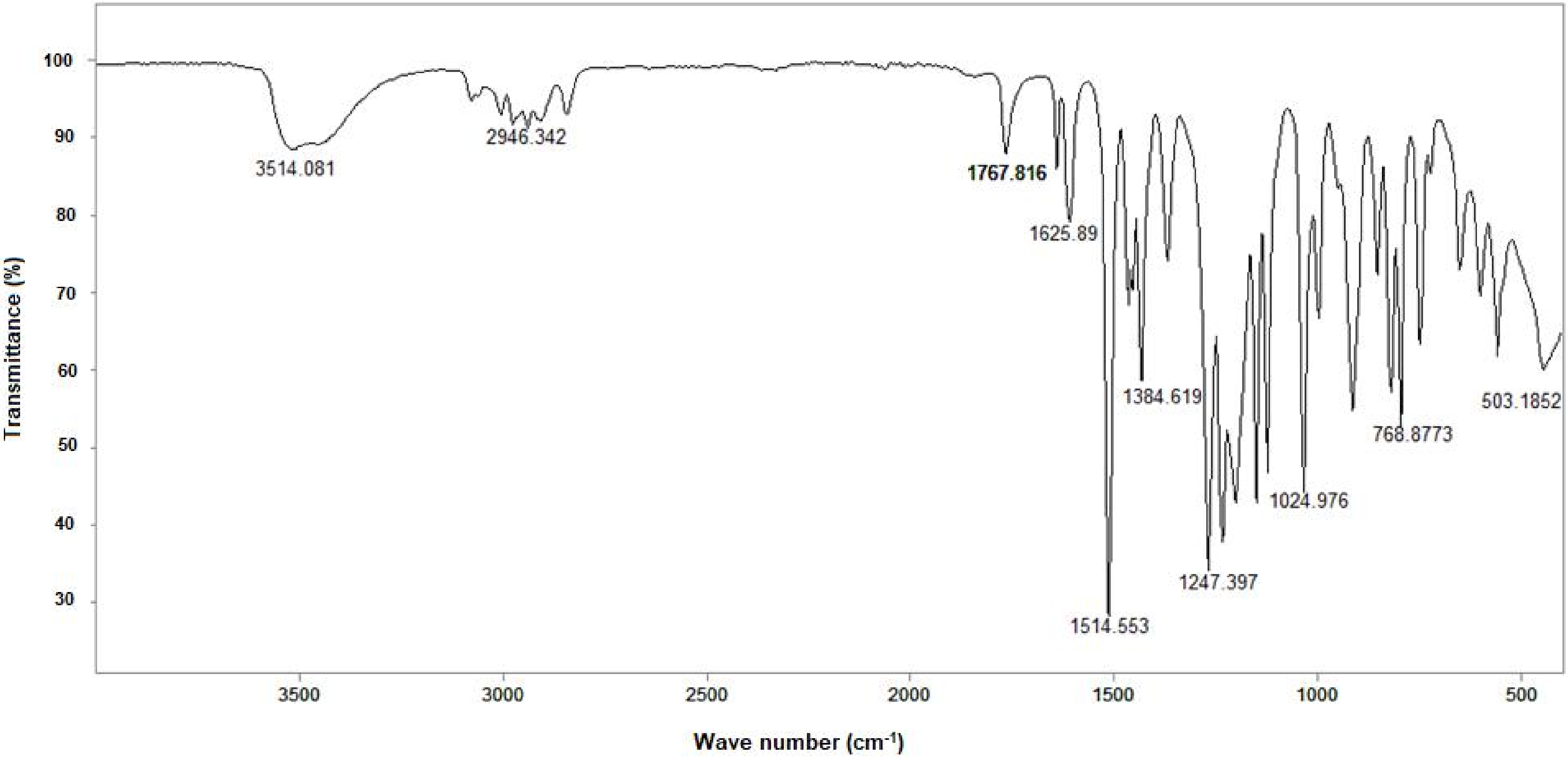

Figure S6. IR (ATR) of clove EO.

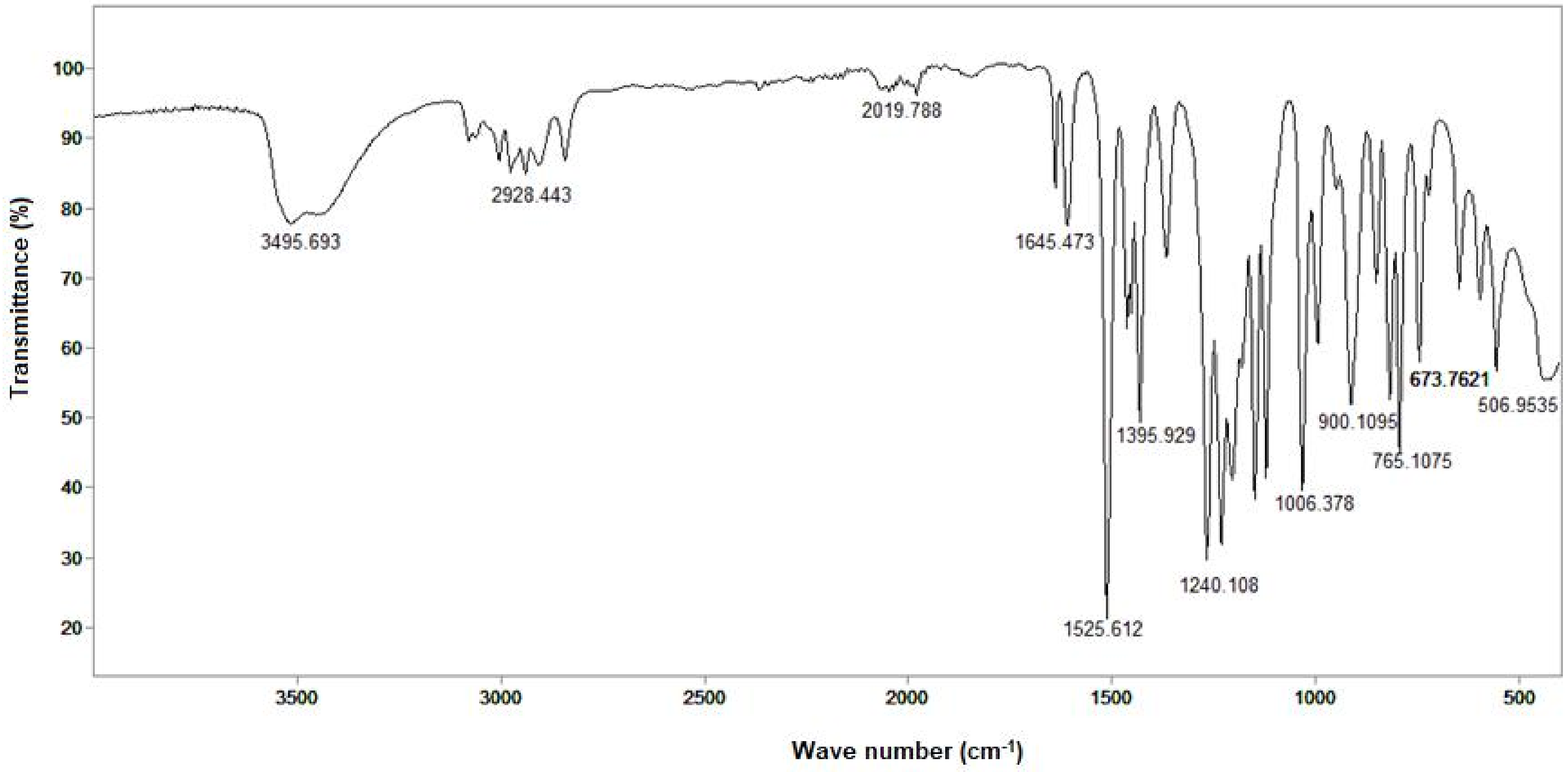

Figure S7. IR (ATR) of pure eugenol.

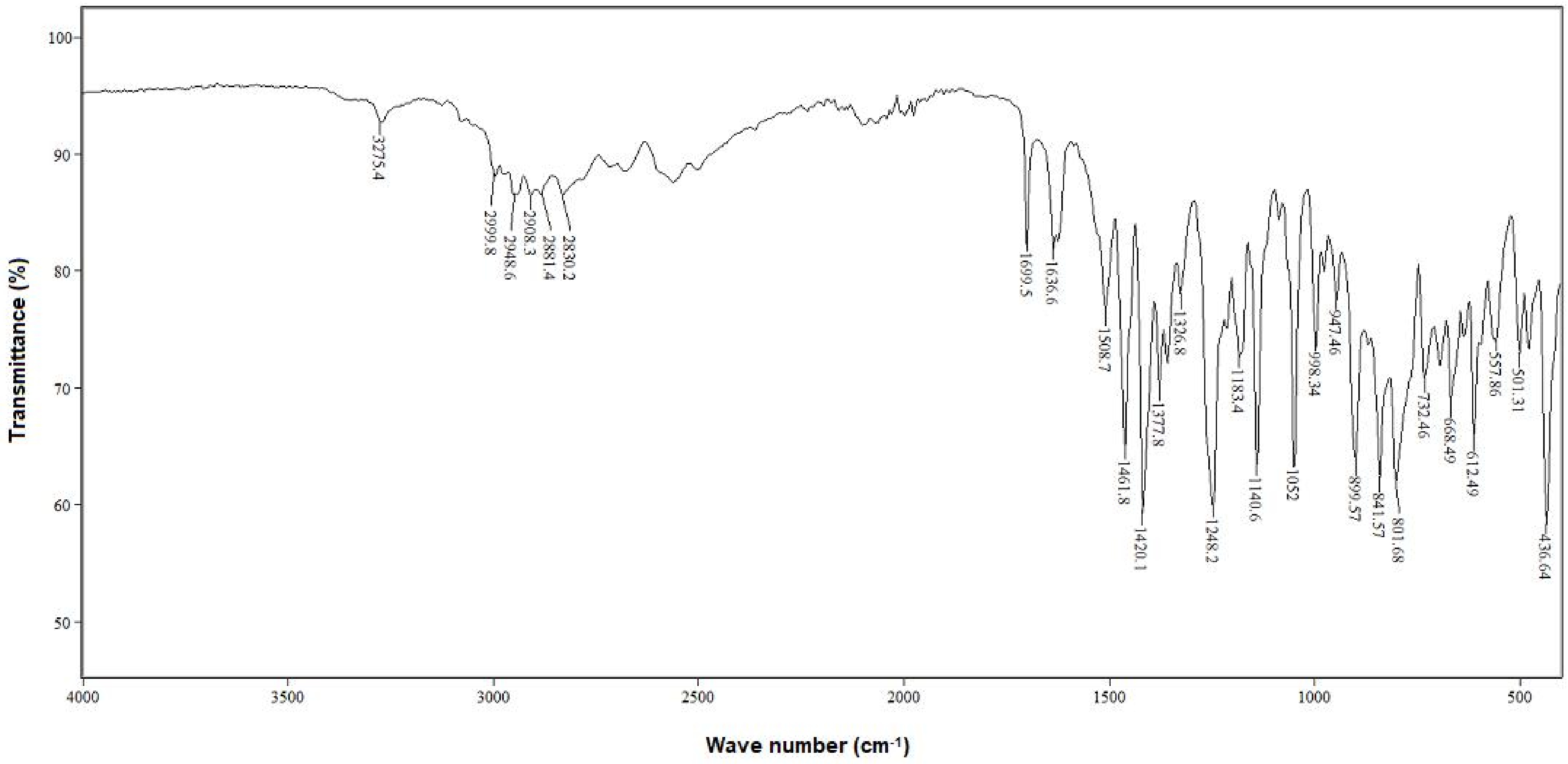

Figure S8. Infrared (ATR) of bis eugenol.

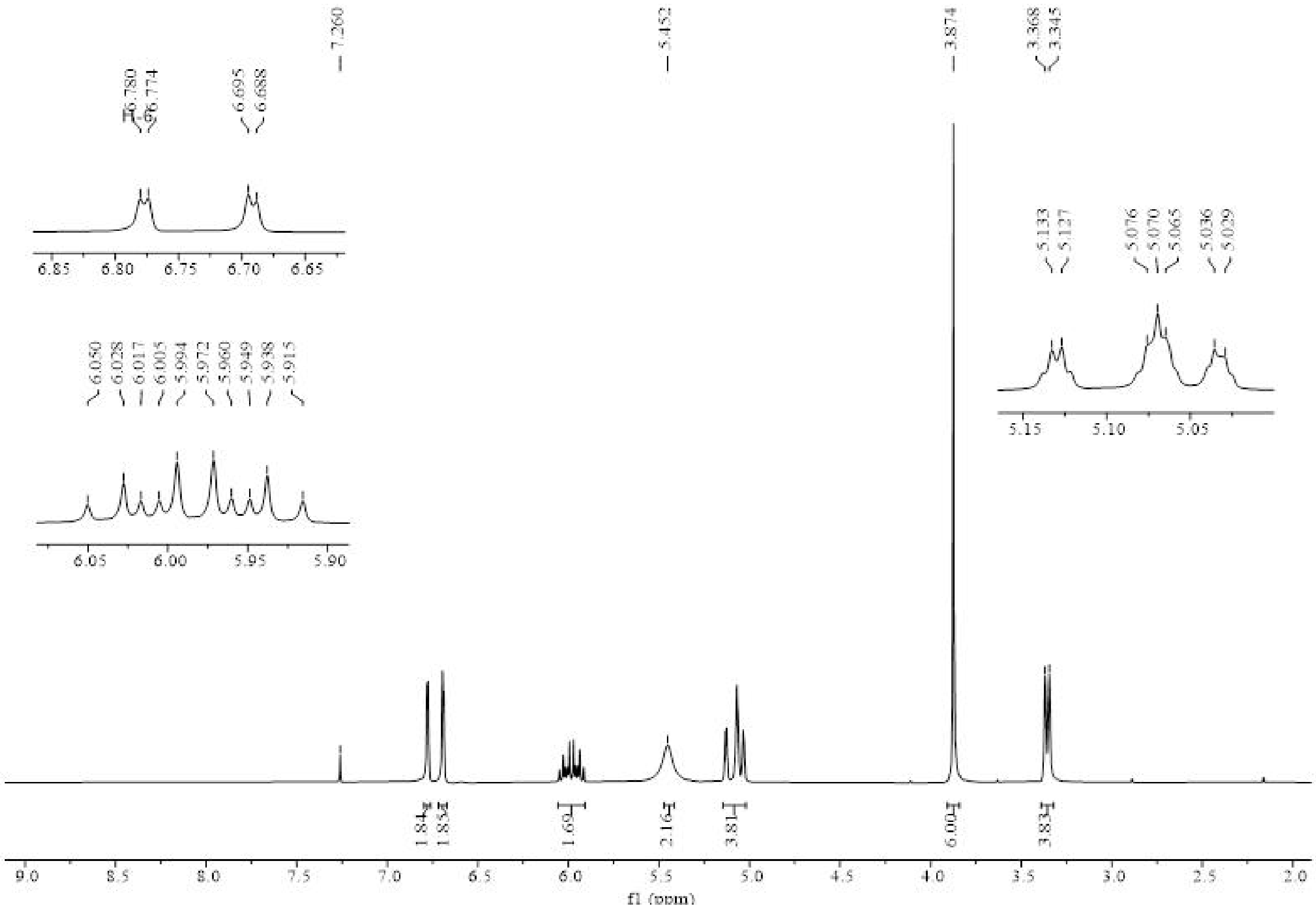

Figure S9. ^{1}H NMR (300 MHz, $CDCl_3$) of bis eugenol.

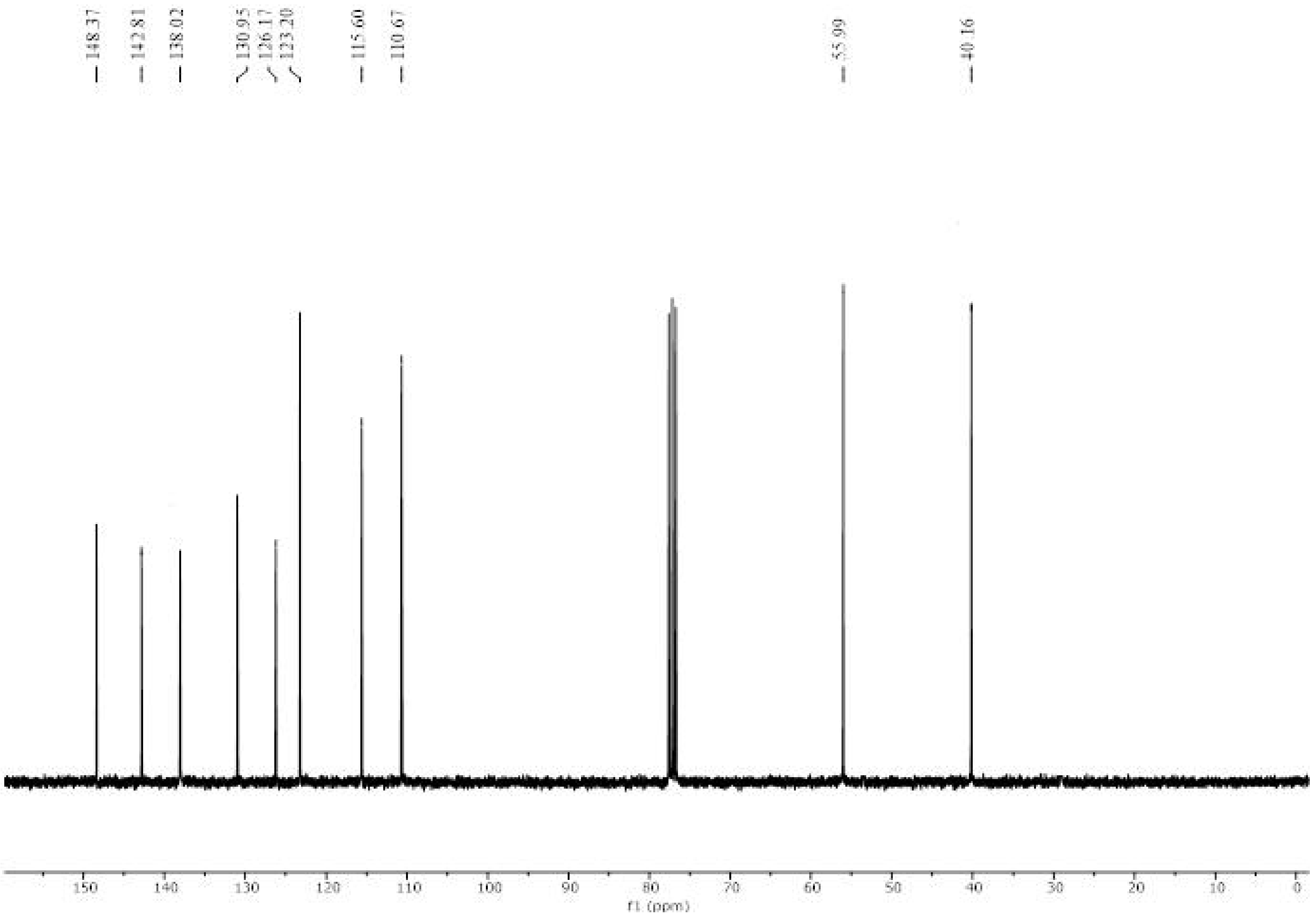

Figure S10 ^{1}H NMR (75 MHz, $CDCl_3$) of bis eugenol.
